# RAS Transformation Requires CUX1-Dependent Repair of Oxidative DNA Damage

**DOI:** 10.1371/journal.pbio.1001807

**Published:** 2014-03-11

**Authors:** Zubaidah M. Ramdzan, Charles Vadnais, Ranjana Pal, Guillaume Vandal, Chantal Cadieux, Lam Leduy, Sayeh Davoudi, Laura Hulea, Lu Yao, Anthony N. Karnezis, Marilène Paquet, David Dankort, Alain Nepveu

**Affiliations:** 1 Goodman Cancer Centre, McGill University, Montreal, Quebec, Canada; 2 Department of Biochemistry, McGill University, Montreal, Quebec, Canada; 3 Department of Biology, McGill University, Montreal, Quebec, Canada; 4 BC Cancer Agency, Centre for Translational and Applied Genomics, Vancouver, British Columbia, Canada; 5 Département de Pathologie et Microbiologie, Faculté de Médecine Vétérinaire, Université de Montréal, St-Hyacinthe, Quebec, Canada; 6 Department of Medicine, McGill University, Montreal, Quebec, Canada; 7 Department of Oncology McGill University, Montreal, Quebec, Canada; St. Jude Children's Research Hospital, United States of America

## Abstract

The Cut homeobox 1 (CUX1) gene is a target of loss-of-heterozygosity in many cancers, yet elevated CUX1 expression is frequently observed and is associated with shorter disease-free survival. The dual role of CUX1 in cancer is illustrated by the fact that most cell lines with CUX1 LOH display amplification of the remaining allele, suggesting that decreased CUX1 expression facilitates tumor development while increased CUX1 expression is needed in tumorigenic cells. Indeed, CUX1 was found in a genome-wide RNAi screen to identify synthetic lethal interactions with oncogenic RAS. Here we show that CUX1 functions in base excision repair as an ancillary factor for the 8-oxoG-DNA glycosylase, OGG1. Single cell gel electrophoresis (comet assay) reveals that *Cux1^+/−^* MEFs are haploinsufficient for the repair of oxidative DNA damage, whereas elevated CUX1 levels accelerate DNA repair. *In vitro* base excision repair assays with purified components demonstrate that CUX1 directly stimulates OGG1's enzymatic activity. Elevated reactive oxygen species (ROS) levels in cells with sustained RAS pathway activation can cause cellular senescence. We show that elevated expression of either CUX1 or OGG1 prevents RAS-induced senescence in primary cells, and that CUX1 knockdown is synthetic lethal with oncogenic RAS in human cancer cells. Elevated CUX1 expression in a transgenic mouse model enables the emergence of mammary tumors with spontaneous activating *Kras* mutations. We confirmed cooperation between Kras^G12V^ and CUX1 in a lung tumor model. Cancer cells can overcome the antiproliferative effects of excessive DNA damage by inactivating a DNA damage response pathway such as ATM or p53 signaling. Our findings reveal an alternate mechanism to allow sustained proliferation in RAS-transformed cells through increased DNA base excision repair capability. The heightened dependency of RAS-transformed cells on base excision repair may provide a therapeutic window that could be exploited with drugs that specifically target this pathway.

## Abstract 2

The base excision repair (BER) that repairs oxidative damage is upregulated as an adaptive response in maintaining tumorigenesis of RAS-transformed cancer cells.

## Author Summary

In the context of tumor development and progression, mutations are believed to accumulate owing to compromised DNA repair. Such mutations promote oncogenic growth. Yet cancer cells also need to sustain a certain level of DNA repair in order to replicate their DNA and successfully proliferate. Here we show that cancer cells that harbor an activated RAS oncogene exhibit heightened DNA repair capability, specifically in the base excision repair (BER) pathway that repairs oxidative DNA damage. RAS oncogenes alone do not transform primary cells but rather cause their senescence—that is, they stop dividing. As such, cellular senescence in this context is proposed to function as a tumor-suppressive mechanism. We show that CUX1, a protein that accelerates oxidative DNA damage repair, prevents cells from senescing and enables proliferation in the presence of a RAS oncogene. Consistent with this, RAS-induced senescence is also prevented by ectopic expression of OGG1, the DNA glycosylase that removes 8-oxoguanine, the most abundant oxidized base. Strikingly, CUX1 expression in transgenic mice enables the emergence of tumors with spontaneous activating *Kras* mutations. Conversely, knockdown of CUX1 is synthetic lethal for RAS-transformed cells, thereby revealing a potential Achilles' heel of these cancer cells. Overall, the work provides insight into understanding the role of DNA repair in cancer progression, showing that while DNA damage-induced mutations promote tumorigenesis, sustained RAS-dependent tumorigenesis requires suppression of DNA damage. The heightened dependency of RAS-transformed cells on base excision repair may provide a therapeutic window that could be exploited with drugs that specifically target this pathway.

## Introduction

Oncogenic potential of RAS signaling is frequently activated in human cancers as a result of point mutations in *RAS* genes or alterations in upstream or downstream signaling proteins (reviewed in [1],[2]). Oncogenic RAS cannot, however, transform primary culture cells alone but requires cooperation with other oncogenic stimulants, a finding that contributed to the concept of multistep tumorigenesis [3]. Subsequent studies have revealed that oncogenic RAS, as well as other oncogenes, cause senescence in both rodent and human primary cells [4]. The concomitant accumulation of p53, p21^CDKN1A^, and p16^INK4a^, together with the finding that proliferation arrest could be bypassed by inactivating the Rb and p53 pathways, promoted the concept that oncogene-induced senescence was a component of the DNA damage response (DDR) that evolved as a tumor suppression mechanism [5]. RAS-induced senescence results from the heightened production of reactive oxygen species (ROS) [6],[7] through increased expression and activity of NADPH oxidases [8],[9]. Among the most deleterious of ROS-induced DNA adducts is 7,8-dihydro-8-oxoguanine (8-oxoG), which can mispair with adenine to cause G-C to T-A transversion mutations [10]. The well-conserved cellular defence system against 8-oxoG involves three main enzymes: MTH1 (MutT in bacteria), a triphosphatase that hydrolyses 8-oxo-dGTP to remove it from the dNTP pool; MYH1 (MutY in bacteria), a DNA glycosylase that catalyzes the excision of adenine from 8-oxoG·A mispairs; and OGG1, a DNA glycosylase that excises 8-oxoG opposite cytosine [11]. The critical role played by 8-oxoG in triggering senescence was demonstrated in experiments where shRNA-mediated knockdown of MTH1 in human skin fibroblasts led to an increase in 8-oxoG levels and caused a senescent phenotype that was associated with several salient features of oncogene-induced senescence including senescence-associated beta-galactosidase (SA-βgal) activity, elevation of p53, p21^CKI^, and p16^INK4a^ proteins, and accumulation of DNA damage [12]. Conversely, MTHI overexpression prevents RAS-induced DDR and the associated premature senescence without affecting ROS levels [13]. In light of these findings, the elevated MTH1 expression in cancers with frequent activating RAS mutations appears to represent a case of nononcogene addiction [14],[15]. This concept posits that tumor cells are acutely dependent on heightened expression or activity of proteins that are not themselves classical oncogenes [16]. High MTH1 expression in tumor cells likely provides a mechanism of adaptation to prevent senescence in response to excessive amount of ROS.

The Cut homeobox 1 (*CUX1*) gene has been implicated in cancer as both a potential tumor suppressor and an oncogene (reviewed in [17]–[19]). On the one hand, *CUX1* is located in the 7q22.1 chromosomal region, which is the target of loss-of-heterozygosity in a number of cancers [20]–[22], and recent studies have pointed to *CUX1* being as the putative tumor suppressor on 7q22.1 [23]–[26]. Yet no mutation has been found in the remaining allele [27]–[30]. The accumulated evidence supports a model of haploinsufficiency whereby the reduced expression of *CUX1* would contribute to the development of the disease [20]. On the other hand, elevated CUX1 expression is frequently observed in various cancers and is associated with shorter disease-free survival ([31]–[33], reviewed in [18]). In particular, the comprehensive molecular characterization of human colon and rectal cancer rated *CUX1* as the fifth most highly relevant gene (*p* value = 3×10^−10^) on a scale showing the correlation between tumor aggressiveness and gene expression/somatic copy number alterations [31]. The dual role of *CUX1* in cancer is illustrated by the fact that most cell lines with LOH of *CUX1* display amplification of the remaining allele (http://cancer.sanger.ac.uk/cancergenome/projects/cell_lines/), suggesting that decreased *CUX1* expression facilitates tumor initiation while increased *CUX1* expression is associated with tumor progression. Indeed, *CUX1* was found in a genome-wide RNAi screen to identify synthetic lethal interactions with oncogenic RAS [34].

*CUX1* encodes two main isoforms that exhibit strikingly different DNA binding and transcriptional properties (reviewed in [17]). The full-length protein, p200 CUX1, contains four evolutionarily conserved DNA binding domains consisting of three Cut repeats (CR1, CR2, and CR3) and a Cut homeodomain (HD) [35]–[38]. p200 CUX1 is an abundant protein that binds to DNA with extremely fast kinetics (rapid “on” and “off” rates) [39]. These properties are not consistent with a role as a classical transcription factor, which are present in low abundance and bind stably to DNA. In mid-G1 phase, 1% to 5% of p200 CUX1 is proteolytically processed by a nuclear cathepsin L isoform to produce the p110 CUX1 isoform [40],[41]. This shorter CUX1 isoform stably interacts with DNA and, depending on promoter context, can function as transcriptional repressor or activator [42],[43]. Another isoform that is aberrantly expressed in human breast cancers, p75 CUX1, was found to exhibit DNA binding and transcriptional properties similar to that of p110 CUX1 [44]. Transcription and cell-based assays demonstrated a role for CUX1 in cell cycle progression and cell proliferation [45],[46], strengthening of the spindle assembly checkpoint [47], DDRs [48], cell migration and invasion [32],[43], resistance to apoptotic signals [33], and dendrite branching and spine development in cortical neurons [49]. The role of CUX1 in many processes was demonstrated using knockdown or genetic inactivation approaches. Knockdown and genetic inactivation approaches have revealed multiples roles of CUX1, but which CUX1 isoform is active in each process could not be established from these methods [32],[33],[49]–[51]. Overexpression studies have demonstrated that p110 CUX1 can stimulate cell cycle progression and cell motility, while the p200 CUX1 isoform is inactive in these assays [43],[45]. Early studies described p200 CUX1 as a transcriptional repressor that functions in precursor cells to down-regulate the expression of genes that become expressed only in terminally differentiated cells [52]–[56]. However, immunohistochemical evidence demonstrates that CUX1 is highly expressed in terminally differentiated cells of several tissues including neurons of the cerebral cortex [33],[49],[51].

The molecular and cellular functions of p200 CUX1 remain to be established. Moreover, while the stimulation of proliferation, cell motility, and resistance to apoptosis provide mechanisms by which CUX1 may contribute to tumorigenicity [32],[33],[43],[45], we have yet to identify a molecular function that could explain the status of CUX1 as a haplo-insufficient tumor suppressor gene. To define and compare the oncogenic potential of CUX1 isoforms without interference from integration site effects or transgene copy number, we used the method of “specific transgenesis” whereby MMTV-p110 CUX1 or MMTV-p200 CUX1 transgenes were integrated by homologous recombination into the *hprt* locus. We previously reported that MMTV-p110 CUX1 transgenic mice develop mammary tumors of various histological types after a long latency [57]. Here we show that MMTV-p200 CUX1 transgenic mice develop mammary tumors with the same penetrance and similar long latency, however a major difference between these transgenic models is that activating mutations in *Kras* were observed in 45% of mammary tumors that developed in MMTV-p200 transgenic mice. Using lentiviral infections in the lung, we confirmed that p200 CUX1 cooperates with activated Kras in tumor formation. Cell-based assays showed that CUX1 accelerates the repair of oxidative DNA damage and prevents RAS-induced senescence in primary fibroblasts. Mechanistic studies revealed that CUX1 functions in base excision repair as an ancillary factor that stimulates the activity of the OGG1 DNA glycosylase. The heightened DNA repair capability conferred by high CUX1 expression is needed to enable the proliferation of RAS-transformed cells in the presence of elevated ROS. On the other hand, the role of CUX1 in base excision repair may explain how haplodeficient expression of CUX1 may contribute to tumor initiation.

## Results

### MMTV-p200 CUX1 Transgenic Mice Develop Late-Onset Mammary Tumors

Characterization of the mammary tumors that developed in the MMTV-p75 and MMTV-p110 CUX1 transgenic mice has previously been reported [57]. To assess and compare the oncogenic potential of p200 CUX1 with that of p75 and p110 CUX1, as previously we used site-specific transgenesis into the *hprt* locus to generate transgenic mice expressing p200 CUX1 under the control of the mouse mammary tumor virus long terminal repeat (Figure S1A) [58]. This strategy minimizes variation from copy number and integration site effects, thus ensuring that each transgene is under the influence of the same regulatory sequences. Transgene expression was detected during pregnancy (Figure S1B). We observed increased ductal branching and budding at 3 months and during pregnancy in transgenic mice (Figure S1C, 3 months, 7.5 and 13.5 days). Moreover, involution appeared to be delayed in transgenic mice (Figure S1D, 1 day involution).

**Figure 1:**
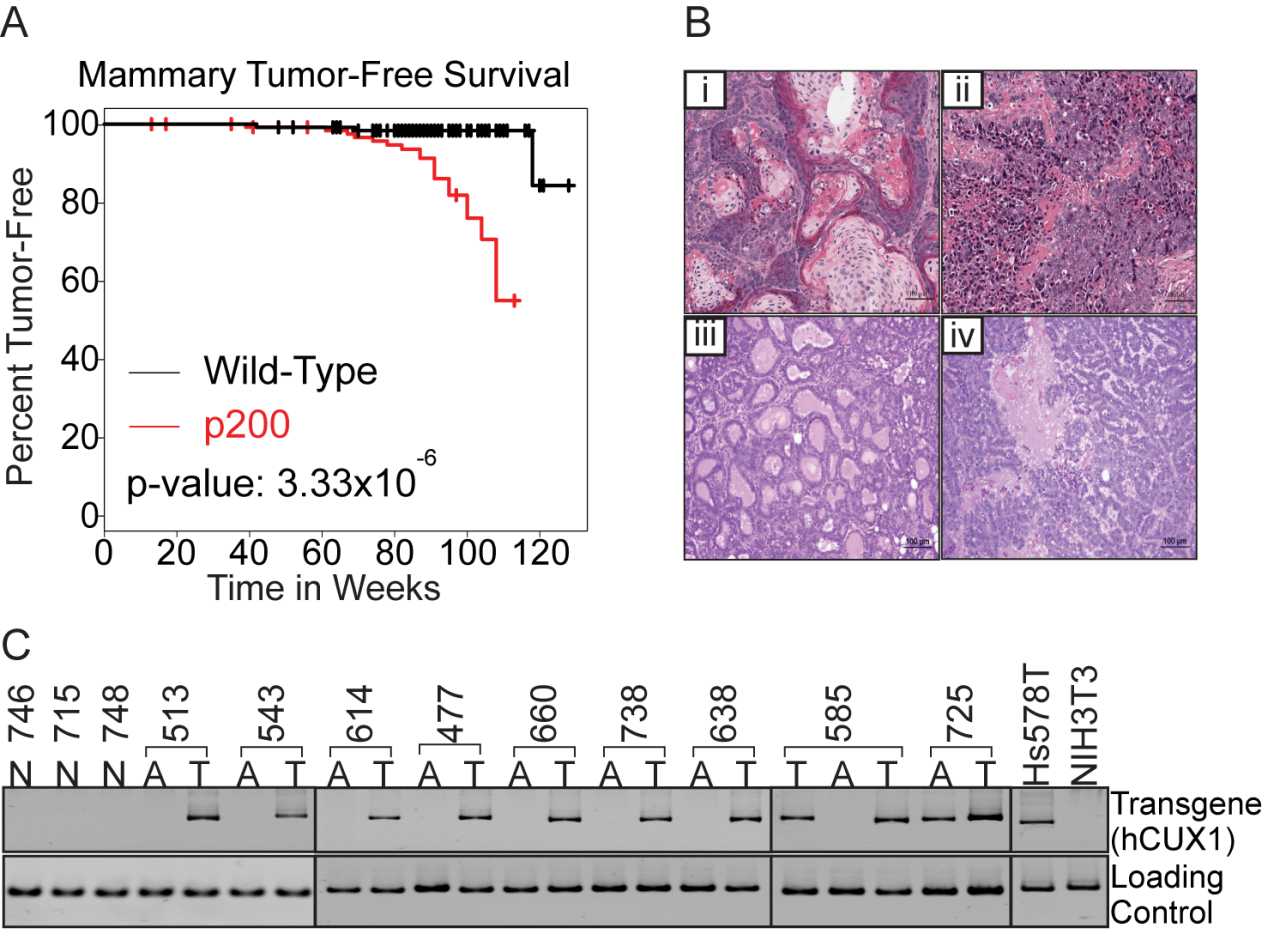
p200 CUX1 transgenic mice develop mammary gland tumors of various histopathologies. (A) Kaplan-Meier survival curves evaluating risk of developing mammary gland tumors in wild-type and p200 CUX1 cohorts. The indicated *p* values were calculated by the log rank test. Using the Cox proportional hazards test, the probability of developing a mammary gland tumor was determined to be 24.7 higher in p200 CUX1 mice (*p* value: 3.33×10^−6^) than in wild-type nontransgenic mice. (B) H&E staining of mammary tumors from p200 CUX1 transgenic mice. Histopathological types were classified as adenosquamous carcinoma (i), solid carcinoma (ii), carcinoma papillary (iii), and carcinoma cribiform (iv). (C) Expression of p200 CUX1 transgenes in mammary tumors (T) and adjacent mammary glands (A) of transgenic mice and normal mammary glands tissues (N) of transgenic mice was analyzed by RT-qPCR. Mouse-specific β2-microglobulin was used as controls for the mammary gland tissues and GAPDH were used for Hs578T and NIH3T3 cell lines.

Cohorts of multiparous MMTV-p200 CUX1 (*n* = 129) and wild-type FVB mice (*n* = 128) were monitored for tumor incidence over 2 years (Figure 1A, Kaplan-Meier plots). Tumors were detected primarily in the mammary glands and lungs (Table 1). Mammary tumors developed in 20.9% of p200 CUX1 transgenic lines as compared to 2.4% of wild-type FVB/N mice (Table 1). Histopathological analysis revealed that mammary tumors were of diverse histopathological types (Figure 1B and Table S1). Adenosquamous carcinoma, solid carcinoma, carcinoma papillary, or carcinoma cribiform were observed (Figure 1B). As expected, CUX1 transgene mRNA was detected in all mammary tumors (Figure 1C). In summary, mammary-specific p200 CUX1 expression increased the incidence of late-onset mammary tumors of various histological types.

**Table 1:** Total number of tumors from wild-type and p200 CUX1 transgenic mice.

Tumor Type	Wild-Type (*n* = 88) [57]	Wild-Type (*n* = 40) (This Study)	p200 CUX1 (*n* = 129) (This Study)
Total number with tumors	19 (21.5%)	5 (12.5%)	61 (47.2%)***
Histiocytic sarcoma	7 (7.9%)	0 (0%)	1 (0.8%)
Mammary gland tumors	3 (3.4%)	0 (0%)	26 (20.9%)***
Lung tumors	10 (1.1%)	3 (7.5%)	26 (20.2%)*
Others	3 (3.4%)	2 (5.0%)	8 (6.2%)

Results from wild-type mice are shown from a previous study as well as from the present study. All wild-type mice were taken into account to calculate the *p* values. Other tumors include hematopoietic tumors, liver tumors, uterine tumors, pancreatic tumors, and intestine tumors.

* *p* value≤0.05;

*** *p* value≤0.0001.

### Elevated Cathepsin L Expression and Proteolytic Processing of p200 CUX1 in Mammary Tumors

We investigated CUX1 expression and DNA binding activity in mammary tumors and normal mammary glands of age-matched transgenic littermates (Figure S2A). Western blot analysis of normal mammary gland tissues revealed a major band of apparent M.W. of 150 kDa and a few other weaker bands of lower M.W. (Figure S2A, lanes 3 and 4). None of these proteins were able to bind to DNA as judged from a Southwestern assay using a CUX1 consensus binding site (Figure S2B, lanes 3 and 4). These results are consistent with those of a previous study that described a C-terminally truncated p150 CUX1 isoform in differentiated mammary glands [59]. In contrast, in tumor samples and in cell lines derived from MMTV-p200 tumors, we observed expression of a CUX1 protein of apparent M.W. of 200 kDa as well as many bands of lower M.W. including a species migrating at 110 kDa (Figure S2A, lanes 1, 2, 5, 6, 7, 8). This species was also recognized by an HA-specific antibody (unpublished data). Moreover, proteins of apparent M.W. of ∼200, ∼140, and ∼110 kDa possessed CUX1-site-specific DNA binding activity as revealed by Southwestern blotting (Figure S2B, lanes 1, 2, 5, 6, 7, 8).

The presence of an active p110 CUX1 species carrying an HA tag in tumor samples and tumor-derived cell lines led us to assess cathepsin L expression in mammary tumors from MMTV-p200, p110, and p75 CUX1 transgenic mice. Cathepsin L mRNA was elevated in the majority of MMTV-p200 CUX1 mammary tumors, however the same was not true for mammary tumors from MMTV-p110 and MMTV-p75 CUX1 transgenic mice (Figure S2D). These results indicate that cathepsin L expression was elevated specifically in MMTV-p200 CUX1 transgenic mice.

### Frequent RAS Pathway Activation in Mammary Tumors from MMTV-p200 CUX1 Mice

Elevated cathepsin L expression has been observed in RAS-transformed cells [60]–[63]. Moreover, retroviral expression of an activated RAS oncogene leads to a rapid increase of both cathepsin L expression and CUX1 proteolytic processing [63]. We therefore performed cDNA sequencing to look for the presence of activating mutations in genes implicated in the RAS pathway. No mutation was found in *Nras*, *Hras*, *Braf*, *Pik3ca*, *Pten*, or *Mek1* (*n* = 11). However, mutations within the *Kras* genes were identified in 5 out of 11 mammary tumors from MMTV-p200 CUX1 transgenic mice (Table 2). These mutations replace a glycine with an aspartic acid at codon 12 (G12D) or a glutamine with a leucine at codon 61 (Q61L). Such mutations were previously reported to maintain KRAS in an active GTP-bound state (reviewed in [2]).

**Table 2:** KRAS mutations in mammary tumors from MMTV-p200 CUX1 transgenic mice.

Type of Samples		Mice No.	KRAS Mutation
Nontransgenic	Normal	5	None
	Normal	13	None
	Normal	747	None
	Normal	753	None
	Normal	754	None
Transgenic	Normal	480	None
	Normal^a^	513	None
	Normal^a^	543	None
	Normal^a^	585	None
	Normal	670	None
	Normal^a^	738	None
	Normal	746	None
	Normal	748	None
	Tumor	123	Q61L
	Tumor	236	Q61L
	Tumor	263	Q61L
	Tumor	284	None
	Tumor	447	None
	Tumor	477	None
	Tumor	513	G12D
	Tumor	543	G12D
	Tumor	585	None
	Tumor	638	None
	Tumor	738	None

a These normal tissues were from the adjacent mammary gland of tumor-bearing mice.

### CUX1 and KRAS^G12V^ Cooperate in Lung Tumor Formation

**Figure 2:**
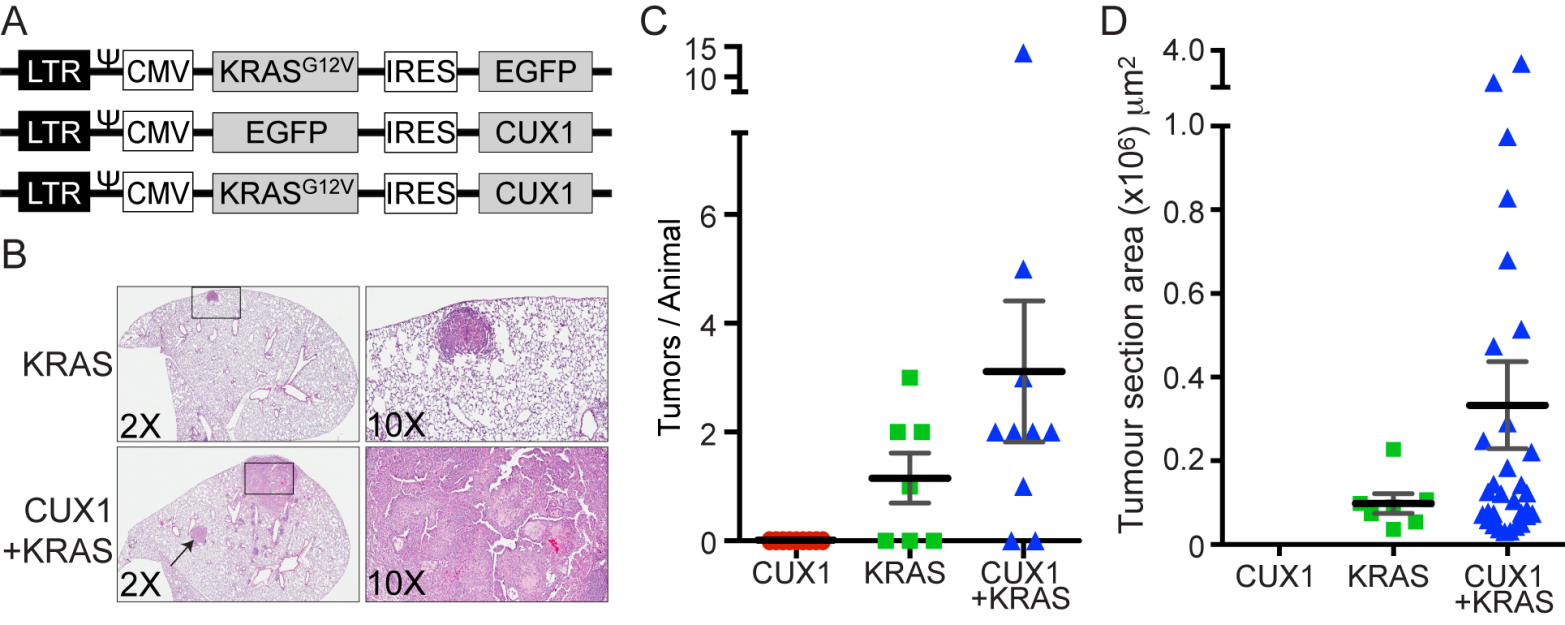
CUX1 and KRAS^G12V^ cooperate in lung tumor formation. (A) Schematic representation of the three lentivirus vectors used for tracheal intubation: KRAS^G12V^–IRES-EGFP, EGFP-IRES-CUX1, and KRAS^G12V^-IRES-CUX1. LTR, 5′-long terminal repeat; Ψ, packaging signal; CMV, cytomegalovirus promoter; IRES, internal ribosomal entry site; EGFP, enhanced green fluorescent protein. (B) FVB/NJ mice were infected via tracheal intubation with individual lentiviruses of identical titer expressing CUX1 (*n* = 9 mice), KRAS^G12V^ (*n* = 7), or CUX1+KRAS^G12V^ (*n* = 10) and lungs were assessed for tumors 18–19 wk later. Tumors were identified following H&E staining of lung sections. Top panels show 2× and 10× magnifications of a grade 1 adenoma caused by KRAS^V12^ expression. Bottom panels show a grade 1 adenoma (arrow) and a grade 2 adenocarcinoma with stromal desmoplasia (box and 10× magnification) caused by CUX1+KRAS^V12^ expression. (C) The graph represents the number of tumors observed per animal. (D) The graph represents the tumor section area calculated as the summed maximal area of all its individual tumors in one or more lung sections for the indicated (number of tumors CUX1 = 0, KRAS^G12V^ = 8, KRAS^G12V^+CUX1 = 31). The difference in tumor burden between KRAS and KRAS+CUX is statistically significant by the Mann–Whitney test, *p* value<0.05.

Forty-five percent of mammary tumors from MMTV-p200 CUX1 transgenic mice sustained activating *Kras* mutations. This finding suggested that CUX1 and activated KRAS cooperate in tumor development. As a rapid assay to test this hypothesis, we infected the lungs of mice with lentiviruses expressing CUX1, KRAS^G12V^, or both CUX1 and KRAS^G12V^ (Figure 2A). CUX1 expression failed to cause tumors to form when assessed at 19 wk postinfection. KRAS^G12V^ expression resulted in an average of two small tumors per mouse developed in four out of seven mice. In contrast 3.6 large tumors per mouse were observed in 8 out of 10 mice that received both CUX1 and KRAS^G12V^ (Figure 2C,D). In summary, although a small number of mice were assessed, the calculated summed area of all tumors indicated that the total tumor burden was 7.5-fold higher in mice infected with a lentivirus expressing both CUX1 and KRAS^G12V^ than in mice that received KRAS^G12V^ alone (*p*<0.05, Mann-Whitney test). Moreover, pathophysiological analysis of these tumors demonstrates that whereas the KRAS^G12V^ mice solely developed grade 1 adenomas (or adenomas with grade 1 nuclear atypia), mice expressing both CUX1 and KRAS^G12V^ developed higher grade adenomas (grades 1 and 2) and one large adenocarcinomas (Figure 2B).

### CUX1 Prevents RAS-Induced Senescence

**Figure 3:**
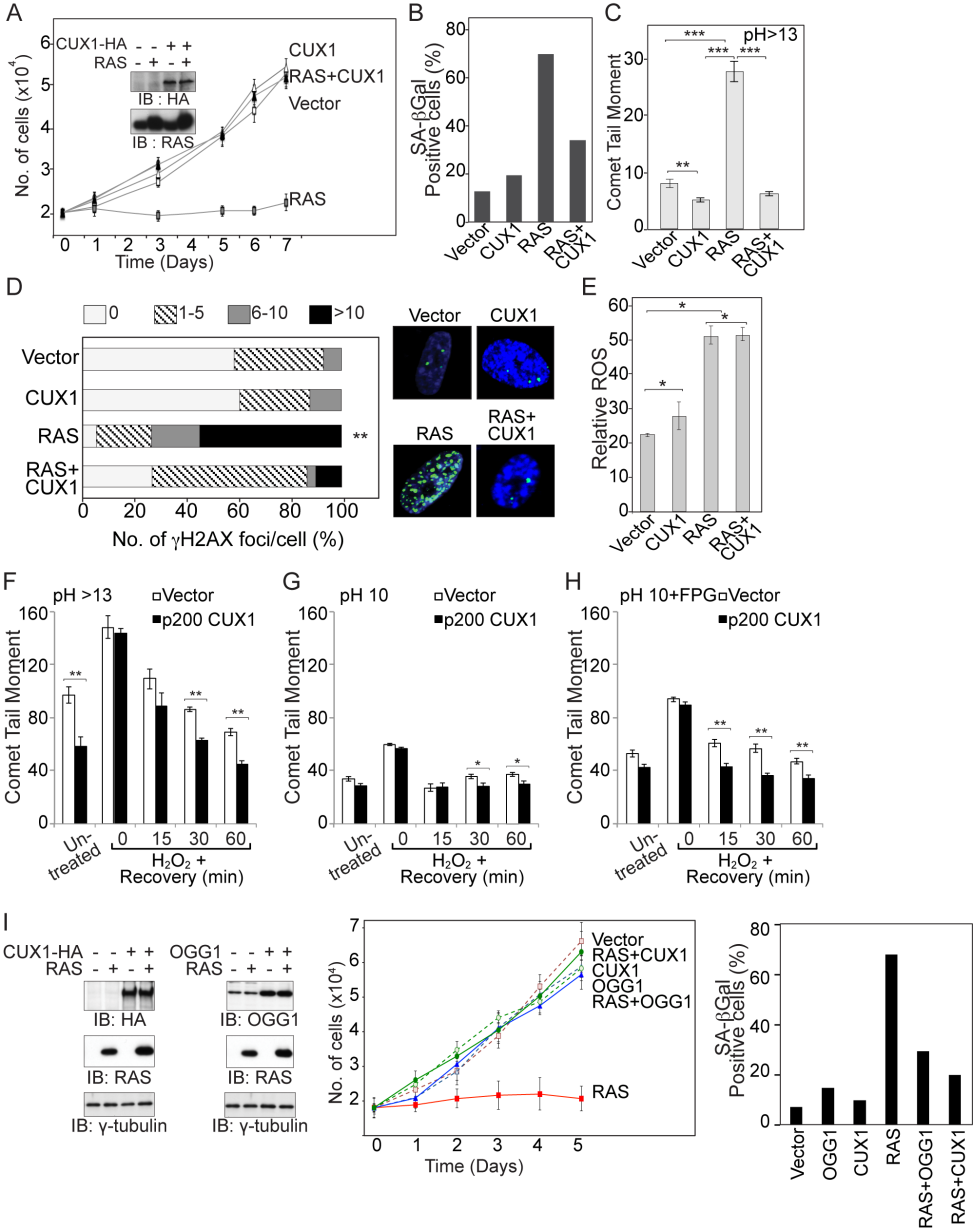
CUX1 prevents RAS-induced cell senescence. (A) IMR90 cells stably expressing p200 CUX1 or carrying an empty vector were infected with a retrovirus expressing HRAS^G12V^ or an empty vector. Following selection, cells were seeded in triplicate and counted for 7 d. The graph is a representative example of three independent experiments. CUX1-HA and HRAS expression were verified by immunoblotting analyses. (B) The percentage of cells exhibiting SA-βgal activity on day 7 was measured. At least 120 cells were analyzed in each case. (C) On day 6 postselection, IMR90 cells were collected and DNA strand breaks quantified by alkaline (pH>13) single-cell gel electrophoresis. The graph is a representative example of three independent experiments. * Indicates *p* value<0.05, **p<0.001, ***p<0.0001 on a student's *t* test. (D) Cells were fixed and stained for γ-H2AX by immunofluorescence. The histograms show the number of cells with 0, 1 to 5, 6 to 10, or more than 10 γ-H2AX foci. At least 80 cells were counted and the percentage of cells with more than 10 foci were used to calculate the *p* value; ** *p*<0.001. (E) Cells were stained with CM-DCF-DA to measure their relative ROS levels via the geometric mean of the fluorescence intensity. (F, G, and H) Cells were treated with 200 µM H_2_O_2_ for 30 min on ice and allowed to recover for 0, 15, 30, and 60 min at 37°C and DNA damage was quantified at pH (pH>13) (F), pH 10 (G), or at pH 10 in the presence of the FPG (H). The graph is a representative example of three independent experiments. (I) IMR90 cells stably expressing p200 CUX1, human OGG1, or carrying an empty vector were infected with a retrovirus expressing HRAS^G12V^ or an empty vector. Proliferation was measured as in (A) and SA-βgal activity was assessed on day 5 as in (B).

Oncogenic RAS cannot itself transform primary culture cells but induces senescence in both rodent and human primary cells [4]. The repeated finding of activating *Kras* mutations in MMTV-p200 CUX1 transgenic mice suggested that the CUX1 transgene provided a terrain in which rare cells that spontaneously acquire an activating *Kras* mutation could proliferate and evolve to become tumorigenic. To test this notion, we examined the proliferation of IMR90 human primary lung fibroblast cells following infection with retroviruses expressing HRAS^G12V^, p200 CUX1, or control virus (Figure 3A). Cells infected with the retrovirus expressing HRAS^G12V^ fail to proliferate and stained positive for senescence-associated β-galactosidase (SA-βgal) activity (Figure 3A,B). Co-expression of p200 CUX1 enabled RAS expressing cells to proliferate normally (Figure 3A) and prevented SA-βgal activity (Figure 3B).

### CUX1 Reduces DNA Damage in RAS-Transformed Cells

RAS-induced senescence has been linked to the accumulation of DNA damage caused by ROS or replicative stress [6],[9],[13]. As reported [9], HRAS^G12V^ expression in IMR90 cells resulted in DNA damage as assessed with single cell gel electrophoresis (comet assays), and immunofluorescence microscopy for phospho-H2AX (γ-H2AX) antibody indicated that higher levels of DNA damage accumulated in IMR90 cells expressing HRAS^G12V^ (Figure 3C,D). Co-expression of p200 CUX1 with HRAS^G12V^, however, completely abrogated the increase in DNA damage and greatly reduced the proportion of cells with more than 5 γ-H2AX foci (Figure 3C,D). We considered two mechanisms by which p200 CUX1 might mitigate DNA damage: it may reduce ROS levels or it may accelerate the repair of oxidative DNA damage. ROS measurements indicated that p200 CUX1 does not reduce but rather increases ROS levels (Figure 3E). Therefore, this is not the mechanism by which CUX1 prevents RAS-induced senescence. To evaluate the effect of p200 CUX1 on oxidative DNA damage repair, IMR90 cells carrying an empty vector or expressing p200 CUX1 were treated with peroxide and allowed to recover for various periods of time before their DNA was assessed for damage in comet assays. Comet assays conducted under alkaline conditions (pH>13) detect double-strand and single-strand breaks, abasic sites, and several types of altered bases that are intrinsically labile at high pH. When performed at pH 10, this assay only detects double- and single-strand breaks. However, at pH 10 addition of DNA glycosylases allows the detection of specific types of altered bases. The formamidopyrimidine DNA-glycosylase (FPG) cleaves the DNA at 8-oxoG (the most abundant oxidized base), formamidopyrimidines, a number of oxidized pyrimidines, and apurinic sites [64]. Comet assays at pH>13 indicated that total DNA damage was repaired more rapidly in cells expressing p200 CUX1 (Figure 3F). Similar results were obtained in REF52 rat embryo fibroblasts (Figure S3). Comet assays at pH 10 in the presence of FPG demonstrated that repair of oxidized bases was accelerated by p200 CUX1 (Figure 3H). Results of comet assays at pH 10 indicated that most additional single-strand break damage was repaired at 15 min (Figure 3G). However, because base excision repair generates single-strand breaks as intermediates, increased damage was observed at 30 and 60 min in the vector cells (Figure 3G). Together these results suggest that elevated CUX1 expression enables RAS-transformed cells to rapidly repair oxidative DNA damage, thereby allowing cells to avoid senescence and continue to proliferate. In support of this notion, expression of ectopic human 8-oxoG DNA glycosylase, OGG1, prevented RAS-induced growth arrest and reduced the proportion of cells exhibiting SA-βgal activity both in IMR90 and REF52 cells (Figures 3I and S3E).

### CUX1 Knockdown Causes an Increase in Oxidative DNA Damage and Is Synthetic Lethal in RAS-Transformed Human Cancer Cell Lines

**Figure 4:**
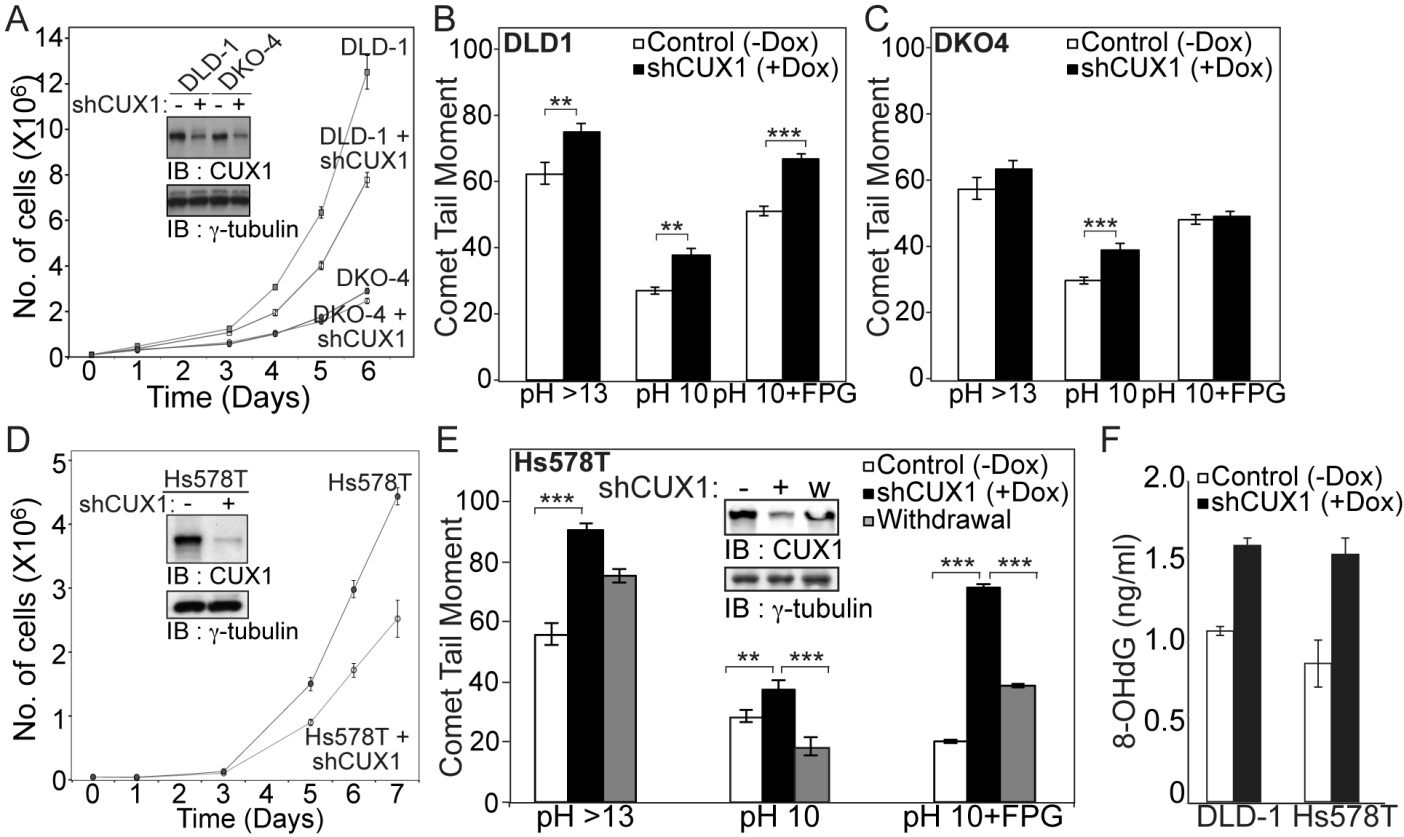
CUX1 knockdown is synthetic lethal for RAS-transformed human cancer cell lines. Lentivirus expressing a doxycycline-inducible shRNA against CUX1 was introduced in a paired cell line DLD-1 [KRAS^G13D^] or DKO-4 [KRAS^WT^] and Hs578T [HRAS^G12D^]. (A) DLD-1 and DKO-4 cells: doxycycline was added to the medium and after 4 d CUX1 protein expression was analyzed by Western blotting. Cells expressing shRNA CUX1 or not were seeded in triplicate and counted daily for 7 d. Each point represents the average ± SD. The graph is a representative example of two independent experiments. (B and C) On day 6, DNA strand breaks were quantified in either alkaline pH (pH 14), pH 10 (detection of single and double strand breaks), or in the presence of the FPG) (detection of single, double, and oxidized purines and formamidopyrimidine). ** *p* value<0.001; *** *p*<0.0001 on a student's *t* test. (D) Hs578T cells: CUX1 protein expression and cell proliferation were analyzed as in Figure 4A. (E) Hs578T cells were cultured in the absence (−) or presence of doxycycline for 4 days (+), followed by a 4-d withdrawal period (w). CUX1 protein expression was analyzed by immunoblotting, and DNA strand breaks for each condition were quantified as described in Figure 4B. * *p* value<0.05; *** *p*<0.0001 on a student's *t* test. (F) 8-OHdG levels were measured in DLD-1 and Hs578T cells expressing shRNA CUX1 or not.

A genome-wide RNAi screen to identify synthetic lethal interactions with the KRAS oncogene identified CUX1 among many other candidates [34],[65]. To validate these findings, we obtained the same pair of cell lines that had been employed in one of these studies [34]. DLD-1 cells encode a KRAS^G13D^ oncogene, whereas the DKO-4 cell line was derived from DLD-1 by inactivating the mutant KRAS allele [66]. Both cell lines were infected with a lentivirus expressing a doxycycline-inducible shRNA targeting CUX1. CUX1 mRNA and protein expression were substantially reduced in both cell lines following treatment with doxycycline (Figures 4A and S4A). CUX1 shRNA significantly reduced cell proliferation in DLD-1 cells, but not in DKO-4 cells (Figure 4A). Reduced proliferation in the presence of CUX1 shRNA was confirmed using a tracking dye to enable measurement of cell division numbers (Figure S4B). Comet assays revealed that DNA damage was increased following the knockdown of CUX1, particularly in DLD-1 cells (Figure 4B,C). In contrast, ROS levels were not significantly increased by CUX1 knockdown (Figure S4C). Similar results were obtained using two distinct CUX1-specific shRNAs as well as an independent pair of cell lines. CUX1 knockdown caused increased oxidative DNA damage and inhibited the proliferation of Hs578T mammary tumor cells, which harbor an HRAS^G12D^ oncogene, but not of Hs578Bst cells, which are normal mammary epithelial cells obtained from the same patient (Figures 4D,E and S4D,E) [67]. DNA damage was also measured in Hs578T cells where we additionally analyzed cells following restoration of CUX1 expression via doxycycline withdrawal. DNA damage increased following CUX1 knockdown and decreased upon CUX1 restoration (Figure 4E). As an adjunct to comet assay analysis to assess DNA damage, we have performed ELISA assays using 8-oxoG–specific antibodies, and found that the 8-oxoG levels in genomic DNA increase following CUX1 knockdown in DLD-1 and Hs578T cells (Figure 4F). These results indicate that CUX1 knockdown causes an increase in oxidative DNA damage and reduces cell proliferation in RAS-transformed cells.

### Genetic Inactivation of One or Two Cux1 Allele(s) Reduces the DNA Repair Efficiency of MEFs

**Figure 5:**
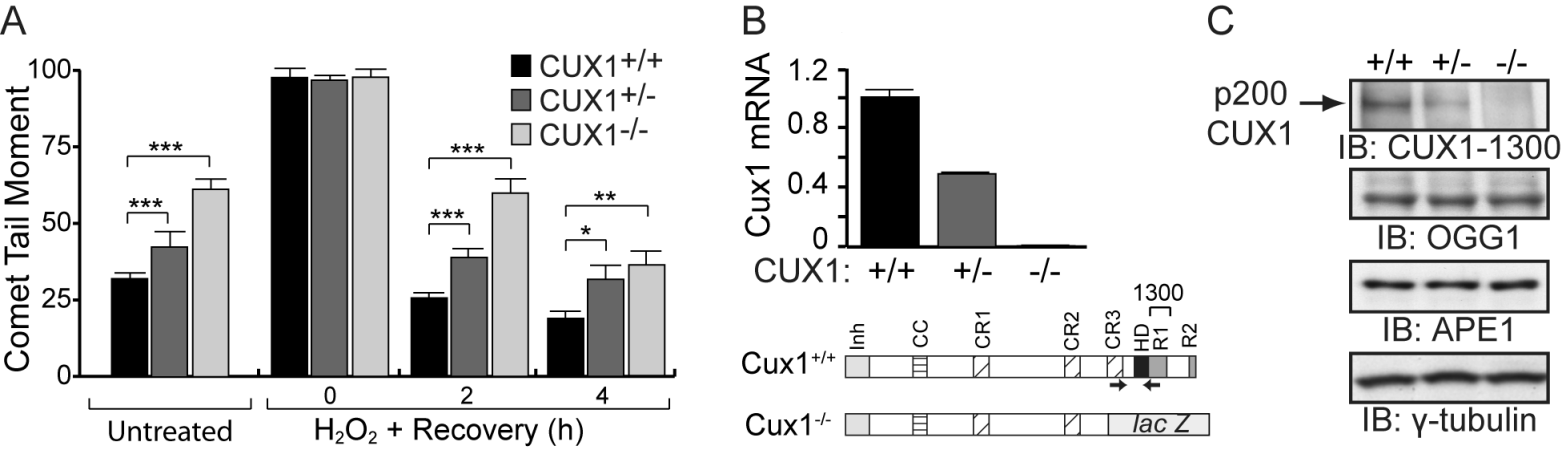
Genetic inactivation of Cux1 reduces the DNA repair efficiency of MEFs. (A) MEFs from Cux1^+/+^, Cux1^+/−^, and Cux1^−/−^ mice were exposed to 10 µm H_2_O_2_ for 20 min on ice, allowed to recover at 37°C for the indicated time. DNA damage before and after treatment was measured by comet assay at pH>13 as in Figure 3F, except that the time course was extended since recovery takes longer in MEFs. Each bar represents the average of at least 30 comets. * *p*<0.05, ** *p*<0.01, *** *p*<0.001. (B) Expression of the wild-type Cux1 gene was analyzed by RT-qPCR. Below is a schematic representation of the wild-type CUX1 protein and the CUX1/lac Z fusion protein present in the knockout cells [51]. Shown at the top are the functional domains: Inh, auto-inhibitory domain; CC, coiled-coil; CR1, CR2, and CR3, Cut repeat 1, 2, and 3; HD, cut homeodomain; R1 and R2, repression domains 1 and 2. Arrows indicate the forward and reverse primers used. (C) Expression of CUX1 (using CUX1–1300 antibody), OGG1, and APE1 was verified by immunoblotting.

We next verified whether genetic inactivation of the *Cux1* gene would impair DNA repair. Mouse embryo fibroblasts (MEFs) from *Cux1* ^+/+^, *Cux1* ^+/−^, and *Cux1* ^−/−^ mice were treated with H_2_O_2_ and submitted to single cell gel electrophoresis (comet) assays after different recovery periods. Prior to treatment, *Cux1* ^−/−^ MEFs exhibited higher levels of DNA damage than wild-type *Cux1^+/+^* MEFs, while heterozygous *Cux1^+/^* ^−^ MEFs displayed intermediate levels of DNA damage (Figure 5A). Consistent with this observation, following treatment with H_2_O_2_ DNA repair was delayed in Cux1^−/−^ MEFs relative to Cux1^+/+^ MEFs (Figure 5A). Interestingly, Cux1^+/−^ MEFs displayed an intermediate phenotype indicating that these cells were haploinsufficient for DNA repair. RT-PCR and immunoblotting analyses demonstrated that Cux1^+/−^ MEFs express intermediate levels of CUX1 (Figure 5B and C). Importantly, OGG1 and APE1 protein expression was similar in the three cell populations (Figure 5C).

### Acceleration of DNA Repair Does Not Require the Transcriptional Functions of CUX1

**Figure 6:**
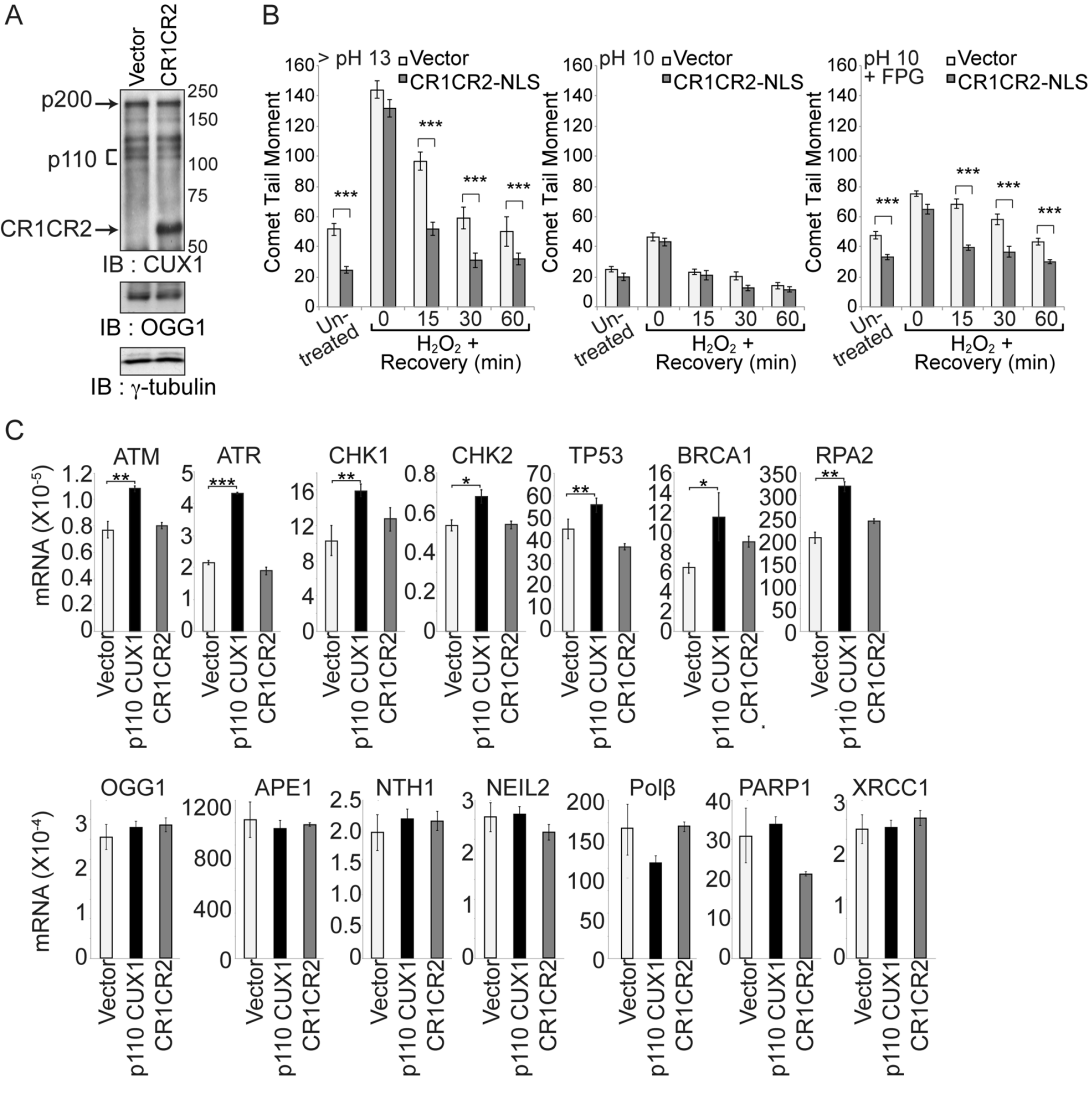
Acceleration of DNA repair does not require the transcriptional functions of CUX1. (A) DLD-1 cells were stably transfected with a plasmid expressing CUX1 CR1CR2 fused to a nuclear localization signal (NLS), or with empty vector (vector). Expression of recombinant CUX1 protein expression and OGG1 levels were analyzed by immunoblotting. (B) DLD-1 cells were exposed to 10 µm H_2_O_2_ for 20 min on ice, allowed to recover at 37°C for the indicated time, and then submitted to Single Cell Gel Electrophoresis (comet assay) to quantify DNA damage as described in Figure 3F,G,H. Each bar represents the average of at least 30 comets. * *p*<0.05, ** *p*<0.01, *** *p*<0.001. (C) RT-PCR analysis was performed to measure mRNA levels of transcriptional targets of p110 CUX1 involved in DDRs and genes involved in base excision repair. Primers are listed in Table S2. All mRNA levels were normalized to glyceraldehyde 3-phosphate dehydrogenase (GAPDH). The values are the mean of three measurements, and error bars represent standard deviation.

The acceleration of DNA repair in cells overexpressing CUX1 could be explained, at least in part, by the role of p110 CUX1 as a transcriptional activator of many genes involved in the DDR [48]. However, we consider it unlikely that such a mechanism could explain the effects of p200 CUX1 overexpression on DNA repair. The full-length CUX1 protein does not function as a transcriptional activator and very little of p200 CUX1 is proteolytic processed to produce p110 CUX1 in cells that are infected with a retrovirus expressing p200 CUX1. On the other hand, the abundance of p200 CUX1 and its extremely fast DNA binding kinetics are compatible with a direct role in DNA repair [39]. These considerations led us to explore the possibility of a nontranscriptional role of CUX1 in DNA repair. To test this hypothesis, we expressed a recombinant protein encompassing the Cut repeats 1 and 2 fused to a nuclear localization signal, CR1CR2-NLS in DLD-1 cells (Figure 6A). Since this protein exhibits very fast DNA binding kinetics and lacks the amino acids required for transcriptional activation, we expected that it would not function as a transcriptional activator [39],[68]. Indeed, gene expression analysis confirmed that transcriptional targets of p110 CUX1 that are involved in DDRs and genes of the base excision repair pathway were not up-regulated in cells stably expressing CR1CR2-NLS (Figure 6C). Despite its inability to activate transcription, CR1CR2-NLS reduced DNA damage in DLD-1 cells (Figure 6B), and accelerated the repair of oxidative DNA damage following treatment with peroxide (Figure 6B). These findings suggest that CUX1 may play a direct role in the repair of oxidized bases.

### CUX1 Plays a Direct Role in DNA Repair by Stimulating the OGG1 DNA Glycosylase

**Figure 7:**
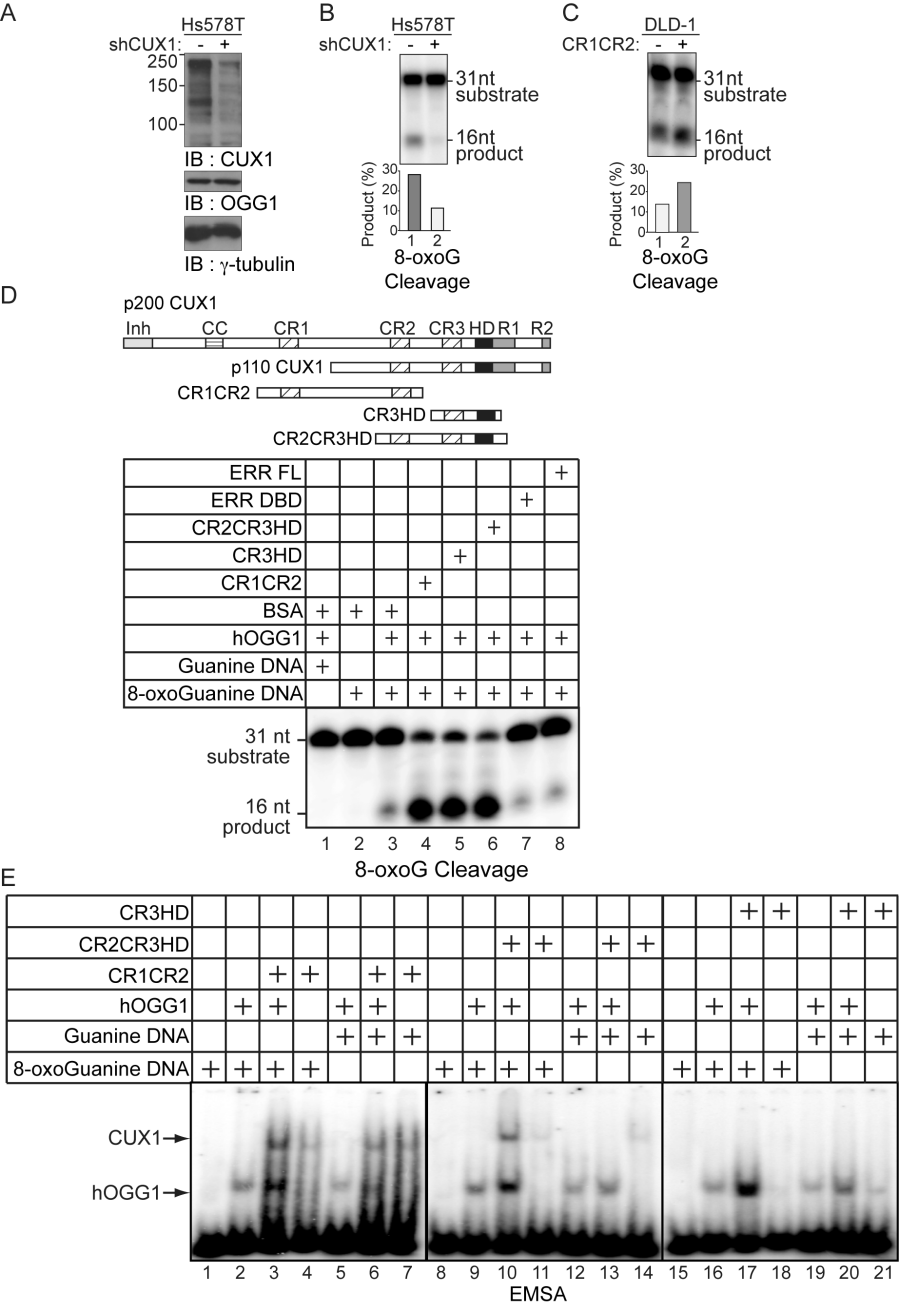
CUX1 stimulates the DNA glycosylase activity of OGG1. (A) Hs578T cells stably carrying a lentivirus expressing a doxycycline inducible CUX1 shRNA were cultured in the absence (−) or presence of doxycycline for 4 d (+). Expression of CUX1 and OGG1 were analyzed by immunoblotting. (B and C) 8-oxoG cleavage assay was conducted using radiolabeled double-stranded oligonucleotides containing an 8-oxoG (map in Figure S5) and 20 µg of whole cell extracts from the indicated cells. (D, Top) Schematic representation of CUX1 proteins used in this study. Shown at the top are the functional domains: Inh, auto-inhibitory domain; CC, coiled-coil; CR1, CR2, and CR3, Cut repeat 1, 2, and 3; HD, cut homeodomain; R1 and R2, repression domains 1 and 2. (D, Bottom) The 8-oxoG cleavage assay was performed using purified human OGG1 and 50 nM of the indicated proteins. ERR FL, full length estrogen-related receptor protein; ERR DBD, DNA binding domain of estrogen-related receptor; CR2CR3HD, CR3HD, and CR1CR2 are CUX1 recombinant proteins described in Figure 6A. (E) Electrophoretic mobility shift assays were performed using oligonucleotides containing an 8-oxoG or an unmodified G and purified OGG1, in the presence or absence of purified CUX1 recombinant proteins, as indicated.

The effects of CUX1 overexpression (Figure 3H) and knockdown (Figure 4B and E) on the repair of oxidized lesions and in particular of 8-oxoG (Figure 4F) led us to investigate this process *in vitro*. It is possible to reproduce a portion of the base excision repair process *in vitro* using cell extracts or purified DNA glycosylases together with double-stranded oligonucleotides containing an 8-oxoG residue. The efficiency of the reaction can be assessed by comparing the signals generated from the substrate and the product after separation on a denaturing gel. This *in vitro* reaction was first performed using whole cell extracts from Hs578T cells before and after induction of CUX1 shRNA. Immunoblot analysis confirmed the CUX1 knockdown, whereas the steady-state level of OGG1 remained unchanged (Figure 7A). Extracts from CUX1 knockdown cells were less efficient at removing 8-oxoG and making a single-strand cut (Figure 7B). In agreement with these results, 8-oxoG cleavage was more efficient with cell extracts from DLD-1 cells expressing CR1CR2-NLS than from cells carrying the empty vector (Figure 7C).

Next, we performed the 8-oxoG cleavage assay using purified human OGG1 in the presence of BSA, various recombinant CUX1 proteins, or another transcription factor as a control (Figure 7D). The enzymatic activity of OGG1 was greatly stimulated by recombinant CUX1 proteins containing one or more Cut repeat domain(s): CR2CR3HD, CR3HD, and CR1CR2. In contrast, OGG1 activity was not stimulated by the full-length estrogen-related receptor alpha (ERRα-FL), ERRα DNA binding domain (ERRα-DBD), or the homeodomain protein B3 (HOXB3) (Figures 7D and S5C). OGG1 activity was stimulated by increasing amounts of CR2CR3HD up to a ratio of 1∶1 (Figure S5E), and time-course analysis showed that equimolar amount of CR2CR3HD accelerated cleavage by OGG1 (Figure S5F). Pull-down assays indicated that CR2CR3HD and OGG1 can interact in the absence of DNA (Figure S5J, lane 3). Importantly, CR2CR3HD alone did not cleave DNA containing an 8-oxoG (Figure S5G, lanes 4 to 7) or an abasic site (Figure S5H, lanes 3 to 7).

To investigate the effect of recombinant CUX1 proteins on the interaction between OGG1 and DNA, electrophoretic mobility shift assays were performed using identical oligonucleotides containing either an 8-oxoG or a normal guanine base. In this case, proteins and DNA were incubated for only 15 min and at 25°C to avoid cleavage of the probe. OGG1, either alone or with a CUX1 protein, generated a stronger retarded complex with the 8-oxoG–containing probe than with the probe containing a normal G (Figure 7E, OGG1 alone: compare lanes 2–5, 9–12, and 6–19; with CUX1, lanes 3–5, 8–10, and 13–15). The retarded complexes formed by OGG1 increased in intensity upon addition of Cut repeat proteins, but their mobility was not affected (Figure 7E, compare lanes 2–3, 9–10, 12–13, 16–17, and 19–20). These results indicate that Cut repeat proteins stimulate the binding of OGG1 to DNA without forming a ternary complex with OGG1 and DNA. The ERR full-length protein like CUX1 stimulated the binding of OGG1 to the 8-oxoG– or G-containing probes (Figure S5I), but in contrast to CUX1 did not increase its catalytic activity (Figure 7D, compare lanes 3 and 7). In summary, results from *in vitro* assays demonstrate that CUX1 stimulates the DNA binding and catalytic activities of OGG1.

## Discussion

The design of MMTV-p75, p110, and p200 CUX1 transgenic mice involved specific integration of each transgene into the same locus (*hprt*) to permit a direct comparison of CUX1 isoform oncogenic potentials without interference from integration site effects or transgene copy number. Another important aspect of our experimental design was to refrain from introducing additional mutations that cause the inactivation of a tumor suppressor or the activation of an oncogene, a manipulation that would have increased tumor burden and shortened the latency period. We reasoned that such an unbiased approach would better recapitulate the process of tumor development as it occurs in humans and therefore reveal significant genetic and epigenetic changes that cooperate with CUX1 overexpression in tumor development. Molecular analysis of mammary tumors from MMTV-p200 CUX1 mice revealed the presence activating *Kras* mutations in 45% of mammary tumors, suggesting that activated RAS and CUX1 cooperate in tumor formation. This hypothesis was verified by performing lentiviral infections in the lung of mice. Indeed, a higher tumor multiplicity, higher grade benign tumors, greater benign tumor burden, and the only adenocarcinoma was observed when CUX1 was co-expressed KRAS^G12V^ (Figure 2). Experiments in primary human and rodent cells suggested that CUX1 increases the number of lung adenomas, when expressed together with KRAS^G12V^, by reducing oxidative DNA damage and preventing cell senescence. Co-expression of CUX1 with HRAS^G12V^ in IMR90 and REF52 primary fibroblasts led to a concomitant decrease in DNA damage (Figures 3C and S3B), DNA damage foci (Figure 3D), and SA-βgal activity (Figure 3B) and enabled RAS-expressing cells to proliferate normally (Figures 3A and S3A). The mechanistic link between efficient oxidative DNA damage repair and continuous proliferation in the presence of a RAS oncogene was confirmed by showing that co-expression of human OGG1 with HRAS^G12V^ reduced SA-βgal activity and promoted rapid proliferation in both IMR90 and REF52 cells (Figures 3I and S3E).

Evidence from a number of studies indicates that senescence can occur in benign tumors. Several senescence-associated markers were found to be expressed in lung adenomas that develop in conditional knock-in mice carrying an endogenous Kras^V12^ oncogene [69]. Similarly, senescence-associated markers were expressed in pancreatic intraductal neoplasias that developed when the Lox-Stop-Lox/Kras^V12^ transgenic mice were crossed with mice that express Cre in the pancreas. These results have been extended to the BRAF^V600E^ knock-in model [70]. Importantly, cell senescence is not restricted to mouse models, but has also been reported in premalignant human colon adenomas [71]–[73], and human benign lesions caused by the BRAF^V600E^ mutation [74], or NF1 inactivation [75]. In summary, many studies indicate that most human and mouse tumor cells stop proliferating and undergo senescence at the premalignant stage, suggesting that it is at this stage that senescence-inducing signals reach sufficient intensity to be effective (reviewed in [76]).

During the course of this study, we became aware that a genomic RNAi screen to identify synthetic lethal interactions with an activated RAS oncogene tentatively identified CUX1 (supplementary table 1 in [34]). We validated the synthetic lethality of CUX1 knockdown in two syngeneic pairs of cell lines that carry or not a RAS oncogene (Figure 4). We noted, however, that proliferation of DKO4 control cells was also slowed down, albeit to a lesser extent, by CUX1 knockdown. This became particularly evident when using the CFSE staining assay, which calculates the proportion of cells having progressed through any number of cell generations (Figure S4B). A negative effect of CUX1 knockdown on DKO4 control cells had also been observed in the original RNAi screen [34]. These findings are consistent with the demonstrated role of CUX1 in cell cycle progression. Notably, Cux1^−/−^ MEFs display a longer G1 phase and proliferate more slowly than their wild-type counterparts [45]. In addition, we cannot exclude that the role of CUX1 in DNA repair is also needed for normal cells to proliferate, as suggested from comet assays at pH 10 with DKO4 cells (Figure 4C). Indeed, a significantly higher proportion of Cux1^−/−^ MEFs exhibit SA-βgal activity in 20% than in 3% oxygen, whereas ectopic expression of p200 CUX1 is able to reduce the proportion of cells that display β-gal activity (Figure S6).

Co-expression of CUX1 enables the proliferation of primary fibroblasts carrying a RAS oncogene (Figures 3 and S3), whereas CUX1 knockdown inhibits cell proliferation in DLD-1-KRAS^G13D^ and Hs578T-HRAS^G12D^ cells (Figure 4). Therefore, CUX1 is not only needed at the start of the transformation process, but persistent CUX1 expression also is required for long-term proliferation of RAS-transformed cells. Importantly, since CUX1 reduces the steady-state level of DNA damage such that checkpoint controls are not activated, the survival and continuous proliferation of tumor cells does not require the inactivation of p19^ARF^/p53 checkpoint controls. Indeed, cell lines established from mammary tumor cells that developed in MMTV-CUX1 transgenic mice display a wild-type p53 and a functional p53/p21^CDKN1A^ axis that can be activated by ionizing radiations (Figure S7).

Most studies investigating RAS-induced senescence in tissue culture originally focused on HRAS, however many studies clearly showed that KRAS can also increase ROS and induce senescence. Overall, the literature suggests that KRAS and HRAS both increase ROS and induce senescence ([77]–[82]; reviewed in [76],[83]). Whether RAS oncogenes must be overexpressed to induce senescence is somewhat controversial and merits some discussion. A Kras^V12^ knock-in was shown to induce senescence in lung adenomas and in pancreatic intraductal neoplasias [82]. As the KRAS oncogene was expressed from its own promoter, it can reasonably be assumed that it was expressed at the physiological level. Another group showed that mouse embryonic fibroblasts expressing the same Kras^V12^ knock-in did not undergo senescence and expression of Kras^V12^ throughout the body failed to induce unscheduled proliferation [84]. As only a fraction of lung bronchiolo-alveolar cells underwent malignant transformation, Kras-induced transformation was proposed to depend on cellular context. Importantly, none of these two studies documented the levels of expression of the Kras^V12^ allele in normal cells or in tumors. The apparent discrepancy between results obtained with similar mouse models could be resolved if we accept the multistep model proposed by the Chodosh group [85]. Using transgenic mice expressing Hras^G12V^ from a doxycycline-inducible promoter, they observed that low levels of Hras^G12V^ expression did not induce senescence or tumorigenicity, but spontaneous up-regulation of Hras^G12V^ expression occurred at low frequency and was associated with senescence and tumor formation. Hence they proposed that Ras-induced tumorigenesis involves at least two steps consisting of the initial activating Ras mutation and then overexpression of the activated Ras allele. We consider it likely that a similar sequence of events occurred with the Kras^G12D^ and Kras^Q61L^ oncogenes that arose spontaneously in our MMTV-p200 CUX1 transgenic mice. Why Kras but not Hras spontaneous mutations were found in tumors from MMTV-CUX1 transgenic mice is not obvious. We note that spontaneous mutations in Kras, but not in Hras, were also found in mammary tumors that developed in transgenic mice expressing a c-Myc transgene in the mammary gland [86]. In the absence of evidence for functional differences between the two oncogenes [87], we are left to speculate that spontaneous mutations in Kras may be more frequent than in Hras, that their expression levels differ, or that a functional interaction exists specifically between Kras and c-Myc or CUX1.

Together our results suggest that elevated CUX1 expression accelerates DNA repair in RAS-transformed cells, thereby mitigating DNA damage to a level that is compatible with continuous cell proliferation. Using FPG DNA glycosylase in comet assays, we were able to show that CUX1 specifically accelerates the repair of 8-oxoG lesions (Figures 3H and 4B and 4E). Ultimately, using purified human OGG1, we found that purified CUX1 proteins containing one or more Cut repeat domains were able to stimulate the enzymatic activity of OGG1, whereas other transcription factors and DNA binding domains were inactive in this assay (Figure 7C). These results demonstrate that CUX1 plays a direct role in the repair of oxidative damage by stimulating the action of OGG1. We cannot, however, exclude the possibility that CUX1 plays additional roles in DNA repair as suggested from the results of comet assays at pH 10 (Figures 4C and 4E and 6B), and the identification of CUX1 as one of the major substrates of PARP1 following treatment with a DNA damaging agent [88].

We noted that the expression of several CUX1 isoforms was elevated in cell lines as compared to the corresponding tumor samples. Two factors may explain this observation. First, tumor samples are obviously heterogeneous and may include cells that express lower CUX1 levels. Secondly, it is likely that cells with higher CUX1 expression are selected in tissue culture. Previous studies demonstrated that the p110 CUX1 isoform can accelerate cell cycle progression and stimulate cell proliferation [45]. Cells expressing more p110 CUX1 would therefore gradually overtake the rest of the population. Moreover, p200 CUX1 itself may confer an advantage in tissue culture by accelerating the repair of oxidative DNA damage.

The discovery that CUX1 can accelerate the function of a DNA glycosylase has important implications in two areas of science. First, the possibility that the function of DNA glycosylases could be facilitated by ancillary factors apparently has not been thoroughly investigated in previous studies. Indeed, there is probably no need for ancillary factors to stimulate base excision repair in short-lived organisms with a small genome. The precedent of CUX1/OGG1 will justify further investigations into distinct classes of DNA binding proteins that participate in the repair of specific types of base damage. Second, to our knowledge, this study describes the first case of nononcogene addiction where transformed cells are dependent for their survival on the heightened activity of a normal protein that plays a direct role in DNA repair. In the context of tumor development and progression, mutations are believed to accumulate owing to compromised DNA repair functions [89]. Therefore, it is generally accepted that defects in DNA repair, whether transient or permanent, contribute to tumor development and progression. Yet, to replicate their DNA and proliferate, cancer cells need DNA repair mechanisms, perhaps even more than do normal cells. Based on our results, we propose that one adaptive response to oxidative stress in RAS-transformed cells is the up-regulation of the pathway that repairs oxidative DNA damage. In support of this notion, we note that among the synthetic lethal interactions with KRAS discovered in the genome-wide RNAi screen conducted by the Elledge group were four other genes that code for proteins involved in base excision repair: NEIL2, XRCC1, Pol beta, and LIG3 [34]. Overall, next to mitotic functions, base excision repair is one of the cellular processes that appears to be essential for the survival of KRAS-transformed cells.

Many studies concur to suggest that CUX1 may function as a haploinsufficient tumor suppressor [20]–[26]. However, none of the reported functions of CUX1 in stimulating cell cycle progression, cell proliferation, cell motility, and resistance to apoptosis is consistent with a role as a tumor suppressor [32],[33],[43],[45]–[47]. In a recent study, the authors claimed that 9 out of 10 unlisted cell cycle genes were inversely correlated with CUX1 expression, thereby implying that its tumor-suppressing function involved the repression of cell cycle genes [25]. Notwithstanding that one cannot judge this claim without knowing the identity of the genes in question, this notion runs counter to a large number of studies from several groups ([17],[90],[91] and references therein). Our results showing that CUX1 knockdown or genetic inactivation of one Cux1 allele impairs DNA repair revealed a molecular activity that could explain how haploinsufficiency of CUX1 may contribute to tumor initiation by promoting the acquisition of mutations in genes and pathways that are involved in the transformation process (Figures 4 and 5). Future experiments should verify whether CUX1 hemizygosity indeed causes an increase in mutations and DNA rearrangements that predispose cells to tumor development. In addition, the fact that most cell lines with LOH of CUX1 display amplification of the remaining allele (http://cancer.sanger.ac.uk/cancergenome/projects/cell_lines/) raises the intriguing possibility that tumor cells with increased CUX1 expression are later selected during tumor progression.

The successful use of a PARP1 inhibitor for the treatment of tumor cells in which BRCA1 or BRCA2 is inactivated has provided a paradigm for the therapeutic exploitation of cancer cell addiction to a specific DNA repair pathway [92]. In the case of BRCA1–2 mutant cancer cells, permanent inactivation of a DNA repair pathway offered the opportunity for therapeutic intervention based on the concept of synthetic lethality [93]. The situation we observe in RAS-transformed cells is different. No obvious DNA repair defect is evident. On the contrary, to proliferate in the presence of elevated ROS and oxidative DNA damage, RAS-transformed cells have adapted by increasing their capacity to repair oxidative DNA damage. Yet this is where the Achilles' heel of these cancer cells may reside. The difference in the frequency of oxidative DNA damage between RAS-transformed cells and normal cells produces an increased dependency on base excision repair which may provide a therapeutic window that could be exploited with drugs that specifically target this pathway.

## Materials and Methods

### Generation of Transgenic Mice

The p200-CUX1 transgenic mice were generated using the human CUX1 cDNA as described in [94], and integrated by site-specific transgenesis into the *Hprt* locus, which resides on the X-chromosome. Two independent lines were backcrossed for at least seven generations with mice of the FVB strain, and as expected, transgene expression in the FVB genetic background was found to be identical in the two lines. To study tumor burden, we generated cohorts of female mice carrying one copy of the transgene on one chromosome X. As a result of random inactivation of one X chromosome in each cell, the transgene would be expected to be expressed in approximately 50% of cells in females.

### Histology, Immunohistochemistry, Immunofluorescence

Hematoxylin and eosin staining and immunohistochemistry were performed as previously described [95]. The following primary antibodies were used: rabbit anti-CUX1 1300 (1∶500) [40] and mouse HA.11 (Covance, 1∶250). Immunofluorescence microscopy for γ-H2AX was performed as previously described [48]. Visualization was done using an Axiovert 200M microscope with an LSM 510 laser module (Zeiss). Images were analyzed using ImageJ64 software.

### Whole Mounts

Inguinal mammary gland number 4 was spread on a glass slide, air dried, fixed overnight in acetone, and stained as previously described [57].

### Reverse Transcription–Quantitative Polymerase Chain Reaction Analysis (RT-qPCR)

Frozen tissue samples were crushed in liquid nitrogen and total RNA was extracted using QIAzol lysis reagent and RNeasy Lipid Tissue Mini Kit (Qiagen) following the manufacturer's instructions. Total RNA from cell lines and RT-qPCR was performed as described by [48]. Primers used are listed in .

### Sequencing for Gene Mutations

Mutations were identified by PCR amplification followed by DNA sequencing. Primers used for amplification and sequencing analysis are listed in Table S2.

### Western Blotting and South-Western

Protein extraction and Western blotting were conducted as described [57]. The following antibodies were used: anti-CUX1 861 and 1300 (1/1,000) [40], anti-HA.11 (Covance, MMS1∶1,000), anti-RAS (BD Transduction, 610001; 1∶1,000), anti-OGG1 (Pierce, PA1-31402; 1∶1,000), anti-APE1 (Santa Cruz, sc-5572, 1∶1,000), anti-p21 (BD Transduction, 556431; 1∶1,000), anti-tubulin (Sigma, T6557; 1∶1,000), and anti-lactate dehydrogenase A (LDHA) (Cell Signaling, 2012; 1∶1,000). South-western blotting was performed using a double-stranded oligonucleotide probe containing the CUX1 consensus-binding site: CGATATCGAT [57].

### Cell Culture and Virus Production

All cells were maintained in Dulbecco's modified minimum essential medium (DMEM, Wisent) supplemented with 10% Fetal Bovine Serum (Tetracycline-free; Invitrogen) and penicillin–streptomycin (Invitrogen). All cells were grown at 37°C, 5% CO_2_, and atmospheric O_2_. Retroviruses were produced using 293VSV cells that were co-transfected with pLXSN-p200 CUX1-HA or pRev/TRE-p110 CUX1-HA and with packaging plasmids pVPack-GP and pVPack-VSV-G (Stratagene). Retrovirus containing HRAS^G12V^ inserted in pBabe (a kind gift from Dr. Scott Lowe) was prepared in the same manner. Lentiviral vectors encoding KRAS^G12V^-ires-eGFP, eGFP-ires-Cux1, and KRAS^G12V^-ires-Cux1 were produced via Gateway recombination into destination vector pLEG R1–R3 [96]. The lentiviral vector expressing human OGG1 was the Precision LentiORF Human OGG1 (with native stop codon), Cat. No. OHS5897-202620019, from Thermo Scientific. Lentiviruses were produced by co-transfecting 293-FT cells with plasmids encoding KRAS-ires-EGFP, EGFP-ires-Cux1, KRAS-ires-Cux1, and pTRIPZ-DoxOn-shCUX1 plasmid (OpenBiosystems; Table S3) and packaging plasmid psPAX2 and envelop plasmid pMD2G. The medium of the transfected cells containing the retrovirus and lentivirus were collected for 5 and 3 d, respectively, starting 48 h posttransfection.

### Lung Infections Via Tracheal Intubation

Concentrated lentiviruses expressing KRAS-ires-EGFP, EGFP-ires-Cux1, and KRAS-ires-Cux1 were titered by infecting 293T cells with 4 µg/ml of polybrene and counting the number of EGFP-positive cells by flow cytometry 72 h postinfection. Relative titers between all viruses were compared by quantifying virion RNA as described in [97]. FVB/NJ mice were anesthetized by intraperitoneal injection of 0.3 mg of avertin per gram of mouse weight. Using tracheal intubation as previously described [98],[99] mice were administered 25 µl of 40 mM sodium caprate to enhance infection followed by 62.5 µl of lentivirus (10^8^ infectious units) 10 min later [100]. During the procedure and up until recovery, the mice were kept on a 37°C pad to prevent hypothermia. The mice were thereafter euthanized at 18 to 19 wk postinfection to harvest the lungs for analysis. Lungs were processed for histology as described in [70]. To quantify both tumor number and tumor burden paraffin, embedded blocks were serial sectioned with 100 µM steps. Tumor section area (µm^2^) was obtained using Aperio ImageScope software after delineating tumor boundaries, using the maximal cross-sectional area obtained for each tumor from different sections.

### Proliferation Analysis

IMR90 and REF52 cells stably expressing either p200-CUX1-HA, p110 CUX1-HA, human OGG1, or carrying an empty vector were plated at a density of 5×10^4^ cells per well in a six-well plate. For the next two days, 2.5 ml of medium containing virus expressing either pBabe HRAS^G12V^ or an empty vector along with 6 µg/ml of polybrene (Roche) were added to the cells and spun at 1,200 *g* for 1 h. At 48 h after infection, cells were selected with appropriate concentration of puromycin. In all experiments, a parallel plate of uninfected cells was completely killed in selective media after 3 d. On the fifth day, hence designated as day 0 in proliferation assays, 2×10^4^ cells per well were seeded in 12-well plates. Each day, cells were trypsinized and counted on a hemocytometer. The medium was replaced every 3 d. Each time point was done in triplicate, and the averages ± standard deviations were calculated. Experiments were repeated three times, and a representative experiment is shown.

### CFSE Staining

Cells were stained using the CellTrace carboxyfluorescein diacetate succinimidyl ester (CFSE) staining cell proliferation kit and were analyzed by flow cytometry with 488-nm excitation and emission filters appropriate for fluorescein, according to the manufacturer's instructions (Molecular Probes/Invitrogen, C34554). CFSE profiles were analyzed using the FlowJo software (Tree Star Software).

### Doxycycline-Inducible shCUX1 Knockdown

DLD-1, DKO-4, Hs578T, and Hs578Bst cells were infected with pTRIPZ-DoxOn-shCUX1 and selected with puromycin. Expression of CUX1-shRNA was induced in the stably infected cells by supplementing the growth media with 1 µg/ml of doxycycline. Cells grown in the absence of doxycycline were used as a control. Knockdown of the CUX1 gene was confirmed by qPCR and Western analysis.

### Intracellular ROS Measurements

Equal number of cells were trypsinized, resuspended in PBS, and incubated with freshly prepared 10M 5-(and-6)-chloromethyl-2′,7′-dichlorofluorescein diacetate (CM-DCF-DA; Molecular Probes/Invitrogen, C6827) for 15 min at 37°C and analyzed by FACS. Geometric mean was determined using FlowJo software (Tree Star Software).

### Single-Cell Gel Electrophoresis

To measure DNA strand breaks, single cell electrophoresis (comet assays) was carried out using precoated slides (Trevigen, MD). Total strand breaks were conducted in alkaline pH as described in [101]. Single and double DNA strand breaks as well as oxidative DNA damage were conducted using FPG enzyme in pH 10 as described by [102]. The slides were stained with propidium iodide and visualized with Axiovert 200M microscope with an LSM 510 laser module (Zeiss). Comet tail moments were measured using the CometScore software (TriTeck Corp). Comet tail moments were scored for at least 50 cells per condition.

### 8-hydroxydeoxyguanosine (8-OHdG) Analysis

DNA was isolated from cells using a Qiagen DNeasy Blood and Tissue kit (Qiagen, Valencia, CA), and DNase-free RNase was used to degrade RNA according to the supplier's protocols with some modifications. Briefly, diethylenetriamine pentaacetic acid (0.1 mM) and ascorbic acid (2 mM) were used to prevent possible background DNA oxidation during the genomic DNA isolation process [103],[104]. The RNA-free DNA obtained was used to determine the 8-OHdG levels using Oxiselect oxidative DNA damage ELISA kit (Cell Biolabs, San Diego, CA).

### *In Vitro* 8-oxoG Cleavage Assay

We obtained 31-mer oligos containing 8-oxoG at position X and complementary oligos with a C opposite X from Integrated DNA Technologies (Coralville, IA). The oligo sequence was 5′-GTGACTACGAGACCTXATGTGACTGAGAGAG- 3′, as previously described [105]. Cleavage reactions with bacterially purified proteins were conducted using 50 nM of proteins and 0.08 U of human OGG1 (New England Biolabs) in 25 mM NaCl, 10 mM Tris (pH 7.5), 1 mM MgCl_2_, 5 mM EDTA (pH 8.0), 5% glycerol, 1 mM of DTT, and 1 pmol of ^32^P radiolabeled double-stranded oligonucleotides containing an 8-oxoG base (Figure S5). Reactions with total cell extracts were performed as described by [106]. In both assays, cleavage reactions were performed at 37°C as previously described. The DNA was loaded on a prewarmed 20% polyacrylamide-urea gel (19∶1) and separated by electrophoresis in Tris-borate and EDTA (TBE; pH 8.0) at constant 20 mAmp. The radiolabeled DNA fragments were visualized by storage phosphor screen (GE Healthcare).

### Electrophoretic Mobility Shift Assay (EMSA)

EMSAs were performed as previously described with the following modifications [40]. Equimolar of bacterially purified proteins were used with or without OGG1 in the reaction together with 60 ng of poly(dI-dC) as a nonspecific competitor DNA. Gels were dried and visualized by storage phosphor screen (GE Healthcare).

## Supporting Information

Figure S1 **Expression of MMTV-p200 CUX1 transgene during development.** (A) A transgene consisting of human p200 CUX1 coding sequences under the control of the mouse mammary tumor virus long terminal repeat (MMTV-LTR) was introduced by specific transgenesis into the Hprt locus on the X chromosome. Functional domains and epitopes recognized by the 861 and 1300 CUX1 antibodies are shown. (B) Immunohistochemical staining of mammary glands from p200 CUX1 mice at different times using 1300 CUX1 and HA antibody. The arrows indicate cells that are positively stained. (C) Whole-mount analysis of mammary glands in wild-type littermates and MMTV-p200 CUX1 from 5-wk- and 3-mo-old virgin and 7.5 and 13.5 d pregnant mice. Five mice per line were analyzed at each time point; representative data are shown. (D) Whole mounts and H&E stains on day 1 and 4 of involution in wild-type littermates and MMTV-p200 CUX1.
(TIF)

Figure S2 **p200 CUX1 protein is expressed and is proteolytically processed in mammary tumor cells of MMTV-p200 CUX1 transgenic mice.** (A) CUX1 protein expression in normal mammary glands tissues (N), mammary tumor tissues (T), and the corresponding tumor cell lines (CLs) was analyzed by Western blotting using CUX1 (861 and 1300) and lactate dehydrogenase A (LDHA) antibodies. (B) DNA binding by CUX1 proteins was analyzed using a Southwestern assay with radiolabeled double-stranded oligonucleotides containing a consensus binding site for all CUX1 isoform: CGATATCGAT. (C) Schematic representation of transgene p200 CUX1 as well as the proteolytic processed isoforms of CUX1: p110 and p150 CUX1. The evolutionarily conserved domains are shown: CC, coiled-coil; CR1, CR2, and CR3, Cut repeat 1, 2, and 3; HD, homeodomain. (D) Cathepsin L mRNA expression was measured by RT-qPCR analysis in mammary tumors from p200, p110, and p75 CUX1 transgenic mice. The results are displayed in a box plot in the right inset. * *p* value≤0.05 using a student's *t* test.
(TIF)

Figure S3 **CUX1 prevents RAS-induced cell senescence in rat fibroblast cells (REF52).** (A) REF52 cells were stably infected with the indicated retroviral vectors expressing HRASG12V, p110 CUX1-HA, or nothing (vector). After 3 d in selective medium, whole-cell extracts were prepared and analyzed by immunoblotting using HA (for CUX1) and RAS antibodies. Following selection, 2×104 cells/cm^2^ were seeded in triplicate and counted 6 d. Each point represents the average ± SD. The graph is a representative example of three independent experiments. (B) On day 6 postselection, REF52 cells were collected and DNA strand breaks quantified by Alkaline Single Cell Gel Electrophoresis at 35 V for 20 min. The graph is a representative example of three independent experiments. * *p* value<0.05, ** *p*<0.001, *** *p*<0.0001 on a student's *t* test. (C) REF52 cells were stained with CM-DCF-DA to measure their relative ROS levels via the geometric mean of the fluorescence intensity. Note that CM-DCFDA is extremely reactive. Therefore, while a comparison between samples within the same experiments is valid, values from this experiment cannot be directly compared with that of Figure 3E. What is consistently observed in IMR90 and REF52 cells, however, is that CUX1 does not reduce ROS levels. Hence, the reduction in DNA damage cannot be explained through an effect on ROS levels. (D) The indicated cells stably expressing CUX1 or carrying an empty retrovirus were treated with 10 µM H2O2 for 30 min and allowed to recover for 0, 15, 30, and 60 min. Note that treatment with H_2_O_2_ was performed at 37°C, which explains that the level of damage in cells expressing p110 CUX1 is already lower at 0 min. DNA strand breaks were quantitated as in Figure 2E, with the exception that cells were electrophoresed for 40 V for 35 min. * *p* value<0.05, ** *p*<0.001, *** *p*<0.0001 on a student's *t* test. (E) REF52 cells stably expressing p200 CUX1, human OGG1, or carrying an empty vector were infected with a retrovirus expressing HRAS^G12V^ or an empty vector. Expression of CUX1-HA, OGG1, and HRAS were verified by immunoblotting. Proliferation was measured and analyzed as in (A). SA-βgal activity was assessed on day 5. At least 120 cells were analyzed in each case.
(TIF)

Figure S4 **Effect of CUX1 knockdown on the number of cell divisions and the level of ROS in DLD-1, DKO-4, Hs578T, and Hs578Bst cells.** A lentivirus expressing a doxycycline inducible shRNA against CUX1 was introduced into DLD-1 (KRAS^G13D^), DKO-4, Hs578T (HRAS^G12D^), and Hs578Bst. (A) CUX1 mRNA was measured by RT-qPCR before and 4 d after induction of CUX1 shRNA expression in DLD-1 and DKO-4 cells. (B) Cell proliferation was measured by staining with CellTrace CFSE. A portion of the population was fixed immediately as the “0” generation. The remaining cells were allowed to proliferate for 6 d in the presence or absence of doxycycline. Cells were fixed and analyzed by flow cytometry. Small peaks within the CFSE profiles represent successive generations, as indicated above the peaks. (C) Cells were stained with CM-DCF-DA to measure their relative ROS via the geometric mean of the fluorescence intensity. (D) CUX1 mRNA and protein expression were investigated by RT-qPCR and immunoblotting analysis. (E) Cell proliferation was measured by staining with CellTrace CFSE as described in (B).
(TIF)

Figure S5 **Probe and purified proteins used in 8-oxoG cleavage assay and EMSA.** (A) Double-stranded oligonucleotides containing an 8-oxoG or an unmodified G at the X position were labeled with 32p-gamma ATP at the 5′ end of the top strand (*) using PNK. Note that these oligonucleotides do not contain a consensus binding site for CUX1. (B) CUX1 and ERR recombinant proteins expressed in bacteria were purified by affinity chromatography, separated by SDS-PAGE, and stained with coomassie blue. (C) 8-oxoG cleavage assay was performed using purified OGG1 and 50 nM of the His-tagged purified HOXB3 and CUX1 proteins. (D) Recombinant proteins expressed in bacteria were purified by affinity chromatography, separated by SDS-PAGE, and stained with coomassie blue. (E) The 8-oxoG cleavage assay was performed using 50 nM OGG1 and increasing amounts of bacterially purified CUX1 CR2CR3HD for 30 min at 37°C. (F) The 8-oxoG cleavage assay was performed at 37°C for the indicated times, using 50 nM OGG1 and 50 nM of bacterially purified CUX1 CR2CR3HD. (G) The 8-oxoG cleavage assay was performed with purified OGG1 together with 50 ng of CUX1 CR2CR3HD or BSA (lanes 1 and 2). As controls, the reaction was carried with increasing amount of CUX1 (CR2CR3HD) alone to verify that this protein does not cleave the 8-oxoG probe. (H) A cleavage assay was performed using a probe containing an abasic site (dspacer) instead of an 8-oxoG to verify that CR2CR3HD alone does not cleave at an abasic site. We used 0.1 U APE1 (NEB) as a positive control. (I) Electrophoretic mobility shift assays were performed using oligonucleotides containing an 8-oxoG or an unmodified G and purified OGG1, in the presence or absence of purified ERR-FL, as indicated. (J) A pull-down assay was performed using purified GST-OGG1 and beads bound to either his-tagged CUX1-CR2CR3HD, his-tagged empty vector, or his-tagged HOXB3 followed by immunoblotting with anti-OGG1.
(TIF)

Figure S6 **Cux1^−/−^ MEFs senesce in 20% oxygen.** (A) MEFs from Cux1^+/+^, Cux1^+/−^, and Cux1^−/−^ mice. Cux1^−/−^ MEFs were stably infected in 3% oxygen with a retrovirus expressing p200 CUX1-HA or an empty vector. Protein expression was verified by immunoblotting analysis using an HA-specific antibody. (B) Following selection, cells were maintained in 3% or 20% oxygen. On day 19, the percentage of cells exhibiting SA-βgal activity was measured. At least 200 cells were analyzed in each condition.
(TIF)

Figure S7 **The p53 checkpoint pathway is functional in mammary tumor cell lines derived from MMTV-p200 CUX1 mice.** Protein expression levels of p21CIP1 in MMTV-p200 CUX1 tumor cell lines were analyzed 4 h after exposure to10 grays of γ-irradiation. C, control cells; γ, γ -irradiated cells.
(TIF)

Table S1 **Distribution of histopathologic types in mammary tumors from p200 CUX1 transgenic mice.**
(DOC)

Table S2 **Primer sequences used for PCR amplification and mutation sequencing analysis.**
(DOC)

Table S3 **Sequences used to design pTRIPz shRNA against CUX1.**
(DOC)

## Abbreviations

8-oxoG: 7,8-dihydro-8-oxoguanine
FPG: formamidopyrimidine DNA-glycosylase
MEFs: mouse embryo fibroblasts
OGG1: 8-oxoguanine glycosylase
ROS: reactive oxygen species
