# Supplementary figures and images for: RAS Transformation Requires CUX1-Dependent Repair of Oxidative DNA Damage
Source: PLoS Biol. 2014 Mar 11;12(3):e1001807. doi: 10.1371/journal.pbio.1001807 (PMC3949673; doi:10.1371/journal.pbio.1001807)

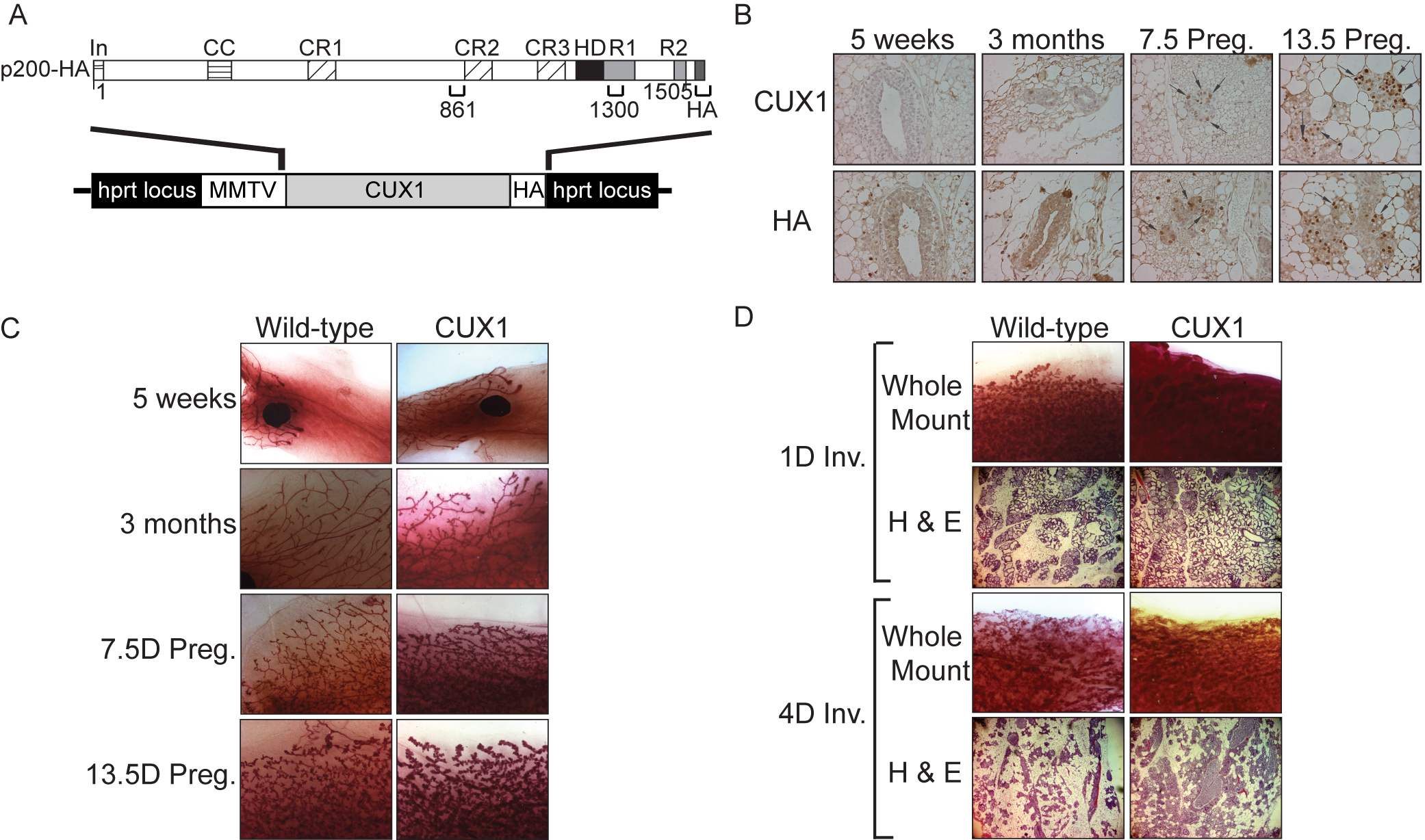

Supplement: Figure S1 — Expression of MMTV-p200 CUX1 transgene during development. (A) A transgene consisting of human p200 CUX1 coding sequences under the control of the mouse mammary tumor virus long terminal repeat (MMTV-LTR) was introduced by specific transgenesis into the Hprt locus on the X chromosome. Functional domains and epitopes recognized by the 861 and 1300 CUX1 antibodies are shown. (B) Immunohistochemical staining of mammary glands from p200 CUX1 mice at different times using 1300 CUX1 and HA antibody. The arrows indicate cells that are positively stained. (C) Whole-mount analysis of mammary glands in wild-type littermates and MMTV-p200 CUX1 from 5-wk- and 3-mo-old virgin and 7.5 and 13.5 d pregnant mice. Five mice per line were analyzed at each time point; representative data are shown. (D) Whole mounts and H&E stains on day 1 and 4 of involution in wild-type littermates and MMTV-p200 CUX1. (TIF) [file pbio.1001807.s001.tif]

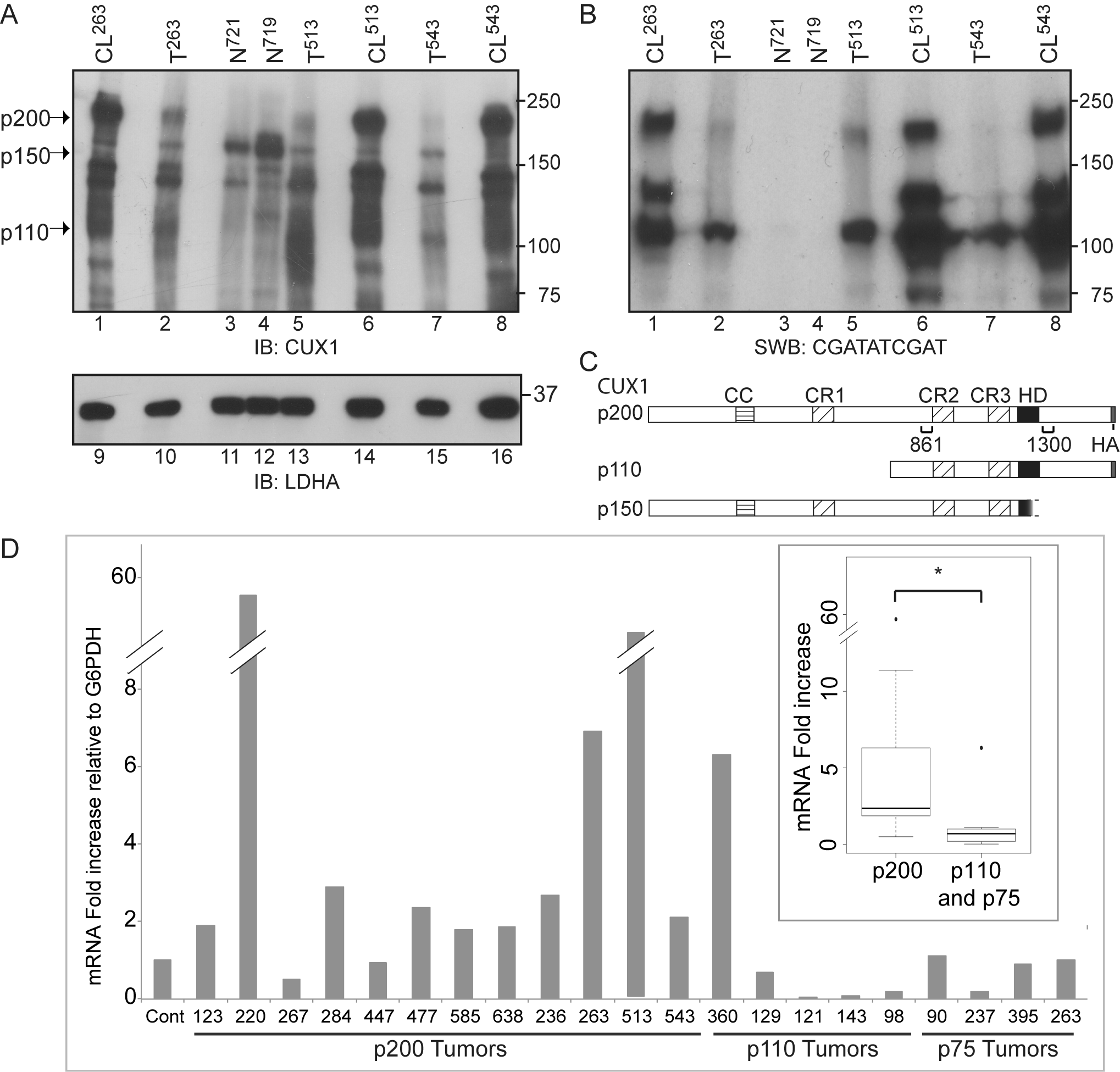

Supplement: Figure S2 — p200 CUX1 protein is expressed and is proteolytically processed in mammary tumor cells of MMTV-p200 CUX1 transgenic mice. (A) CUX1 protein expression in normal mammary glands tissues (N), mammary tumor tissues (T), and the corresponding tumor cell lines (CLs) was analyzed by Western blotting using CUX1 (861 and 1300) and lactate dehydrogenase A (LDHA) antibodies. (B) DNA binding by CUX1 proteins was analyzed using a Southwestern assay with radiolabeled double-stranded oligonucleotides containing a consensus binding site for all CUX1 isoform: CGATATCGAT. (C) Schematic representation of transgene p200 CUX1 as well as the proteolytic processed isoforms of CUX1: p110 and p150 CUX1. The evolutionarily conserved domains are shown: CC, coiled-coil; CR1, CR2, and CR3, Cut repeat 1, 2, and 3; HD, homeodomain. (D) Cathepsin L mRNA expression was measured by RT-qPCR analysis in mammary tumors from p200, p110, and p75 CUX1 transgenic mice. The results are displayed in a box plot in the right inset. * p value≤0.05 using a student's t test. (TIF) [file pbio.1001807.s002.tif]

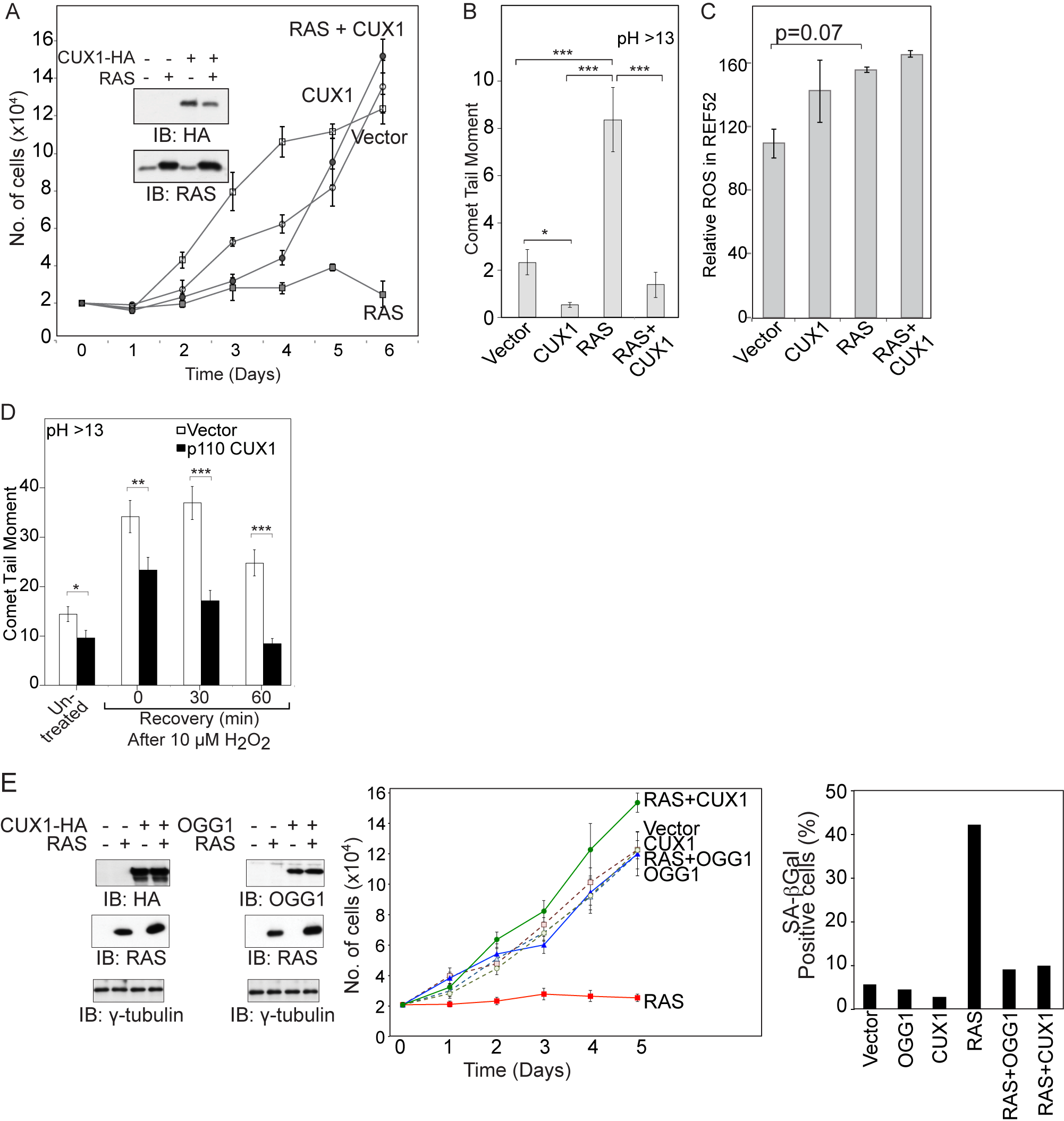

Supplement: Figure S3 — CUX1 prevents RAS-induced cell senescence in rat fibroblast cells (REF52). (A) REF52 cells were stably infected with the indicated retroviral vectors expressing HRASG12V, p110 CUX1-HA, or nothing (vector). After 3 d in selective medium, whole-cell extracts were prepared and analyzed by immunoblotting using HA (for CUX1) and RAS antibodies. Following selection, 2×10^4^ cells/cm2 were seeded in triplicate and counted 6 d. Each point represents the average ± SD. The graph is a representative example of three independent experiments. (B) On day 6 postselection, REF52 cells were collected and DNA strand breaks quantified by Alkaline Single Cell Gel Electrophoresis at 35 V for 20 min. The graph is a representative example of three independent experiments. * p value<0.05, ** p<0.001, *** p<0.0001 on a student's t test. (C) REF52 cells were stained with CM-DCF-DA to measure their relative ROS levels via the geometric mean of the fluorescence intensity. Note that CM-DCFDA is extremely reactive. Therefore, while a comparison between samples within the same experiments is valid, values from this experiment cannot be directly compared with that of Figure 3E. What is consistently observed in IMR90 and REF52 cells, however, is that CUX1 does not reduce ROS levels. Hence, the reduction in DNA damage cannot be explained through an effect on ROS levels. (D) The indicated cells stably expressing CUX1 or carrying an empty retrovirus were treated with 10 µM H2O2 for 30 min and allowed to recover for 0, 15, 30, and 60 min. Note that treatment with H2O2 was performed at 37°C, which explains that the level of damage in cells expressing p110 CUX1 is already lower at 0 min. DNA strand breaks were quantitated as in Figure 2E, with the exception that cells were electrophoresed for 40 V for 35 min. * p value<0.05, ** p<0.001, *** p<0.0001 on a student's t test. (E) REF52 cells stably expressing p200 CUX1, human OGG1, or carrying an empty vector were infected with a retrovirus expressing HRASG12V [file pbio.1001807.s003.tif]

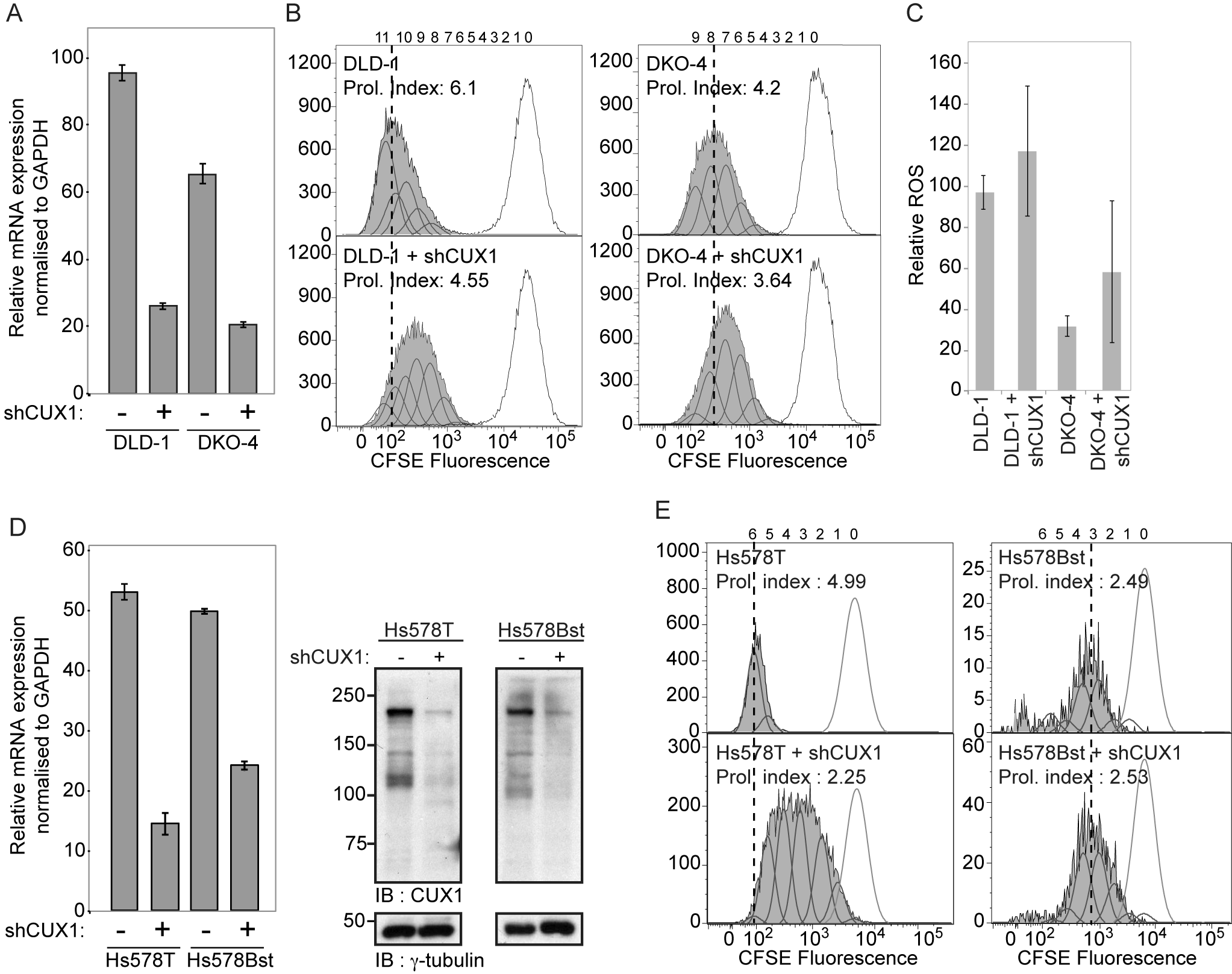

Supplement: Figure S4 — Effect of CUX1 knockdown on the number of cell divisions and the level of ROS in DLD-1, DKO-4, Hs578T, and Hs578Bst cells. A lentivirus expressing a doxycycline inducible shRNA against CUX1 was introduced into DLD-1 (KRASG13D), DKO-4, Hs578T (HRASG12D), and Hs578Bst. (A) CUX1 mRNA was measured by RT-qPCR before and 4 d after induction of CUX1 shRNA expression in DLD-1 and DKO-4 cells. (B) Cell proliferation was measured by staining with CellTrace CFSE. A portion of the population was fixed immediately as the “0” generation. The remaining cells were allowed to proliferate for 6 d in the presence or absence of doxycycline. Cells were fixed and analyzed by flow cytometry. Small peaks within the CFSE profiles represent successive generations, as indicated above the peaks. (C) Cells were stained with CM-DCF-DA to measure their relative ROS via the geometric mean of the fluorescence intensity. (D) CUX1 mRNA and protein expression were investigated by RT-qPCR and immunoblotting analysis. (E) Cell proliferation was measured by staining with CellTrace CFSE as described in (B). (TIF) [file pbio.1001807.s004.tif]

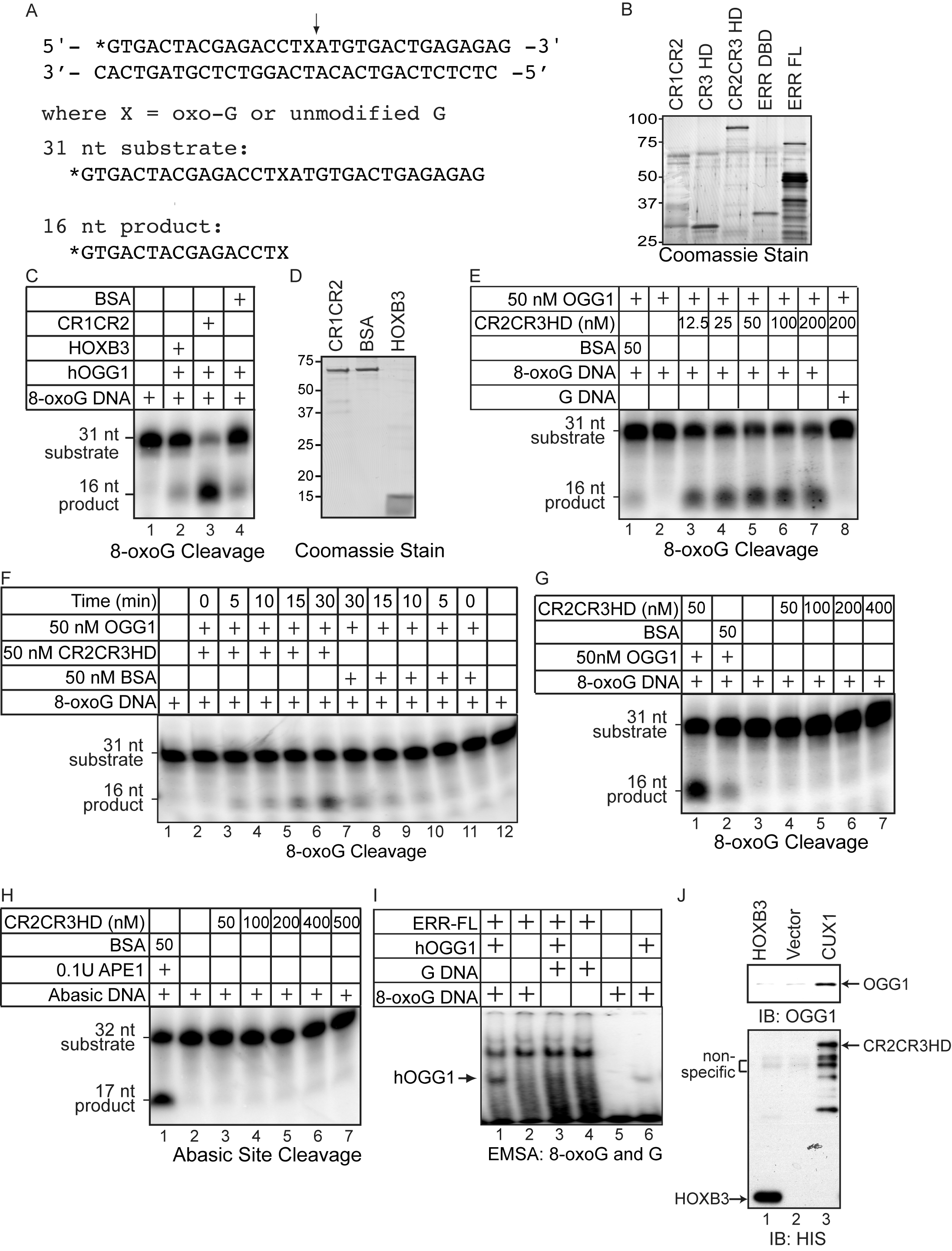

Supplement: Figure S5 — Probe and purified proteins used in 8-oxoG cleavage assay and EMSA. (A) Double-stranded oligonucleotides containing an 8-oxoG or an unmodified G at the X position were labeled with 32p-gamma ATP at the 5′ end of the top strand (*) using PNK. Note that these oligonucleotides do not contain a consensus binding site for CUX1. (B) CUX1 and ERR recombinant proteins expressed in bacteria were purified by affinity chromatography, separated by SDS-PAGE, and stained with coomassie blue. (C) 8-oxoG cleavage assay was performed using purified OGG1 and 50 nM of the His-tagged purified HOXB3 and CUX1 proteins. (D) Recombinant proteins expressed in bacteria were purified by affinity chromatography, separated by SDS-PAGE, and stained with coomassie blue. (E) The 8-oxoG cleavage assay was performed using 50 nM OGG1 and increasing amounts of bacterially purified CUX1 CR2CR3HD for 30 min at 37°C. (F) The 8-oxoG cleavage assay was performed at 37°C for the indicated times, using 50 nM OGG1 and 50 nM of bacterially purified CUX1 CR2CR3HD. (G) The 8-oxoG cleavage assay was performed with purified OGG1 together with 50 ng of CUX1 CR2CR3HD or BSA (lanes 1 and 2). As controls, the reaction was carried with increasing amount of CUX1 (CR2CR3HD) alone to verify that this protein does not cleave the 8-oxoG probe. (H) A cleavage assay was performed using a probe containing an abasic site (dspacer) instead of an 8-oxoG to verify that CR2CR3HD alone does not cleave at an abasic site. We used 0.1 U APE1 (NEB) as a positive control. (I) Electrophoretic mobility shift assays were performed using oligonucleotides containing an 8-oxoG or an unmodified G and purified OGG1, in the presence or absence of purified ERR-FL, as indicated. (J) A pull-down assay was performed using purified GST-OGG1 and beads bound to either his-tagged CUX1-CR2CR3HD, his-tagged empty vector, or his-tagged HOXB3 followed by immunoblotting with anti-OGG1. (TIF) [file pbio.1001807.s005.tif]

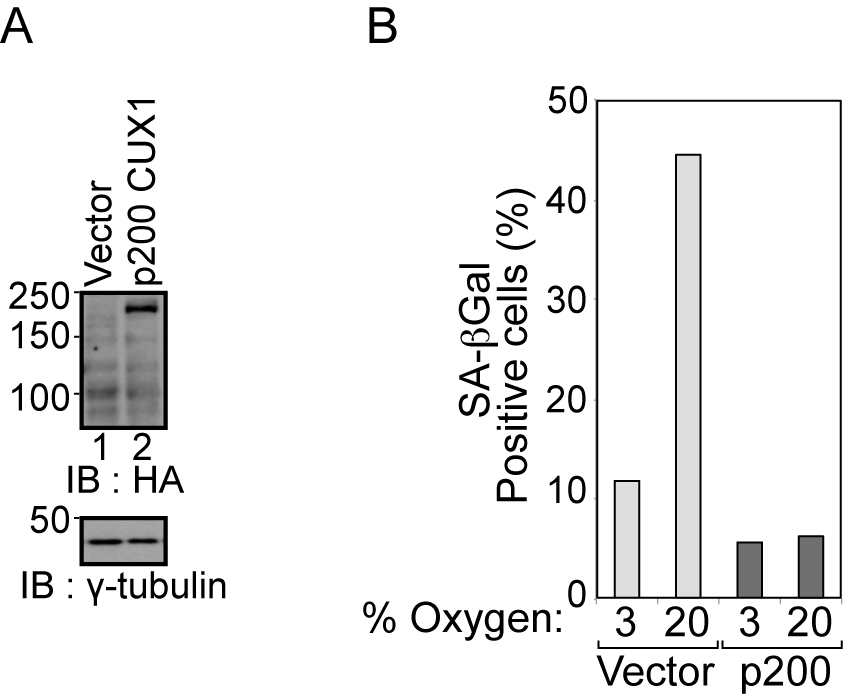

Supplement: Figure S6 — Cux1−/− MEFs senesce in 20% oxygen. (A) MEFs from Cux1+/+, Cux1+/−, and Cux1−/− mice. Cux1−/− MEFs were stably infected in 3% oxygen with a retrovirus expressing p200 CUX1-HA or an empty vector. Protein expression was verified by immunoblotting analysis using an HA-specific antibody. (B) Following selection, cells were maintained in 3% or 20% oxygen. On day 19, the percentage of cells exhibiting SA-βgal activity was measured. At least 200 cells were analyzed in each condition. (TIF) [file pbio.1001807.s006.tif]

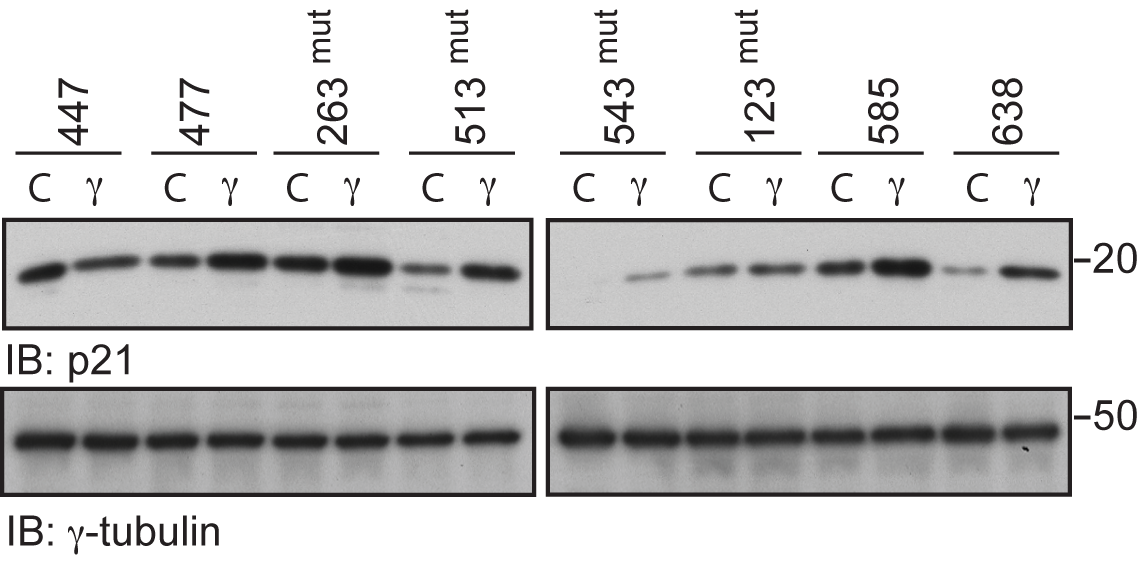

Supplement: Figure S7 — The p53 checkpoint pathway is functional in mammary tumor cell lines derived from MMTV-p200 CUX1 mice. Protein expression levels of p21CIP1 in MMTV-p200 CUX1 tumor cell lines were analyzed 4 h after exposure to10 grays of γ-irradiation. C, control cells; γ, γ -irradiated cells. (TIF) [file pbio.1001807.s007.tif]
