# Supplementary material for: RAS Transformation Requires CUX1-Dependent Repair of Oxidative DNA Damage
Source: PLoS Biol. 2014 Mar 11;12(3):e1001807. doi: 10.1371/journal.pbio.1001807 (PMC3949673; doi:10.1371/journal.pbio.1001807)
Supplement: Table S1 — Distribution of histopathologic types in mammary tumors from p200 CUX1 transgenic mice. (DOC) [file pbio.1001807.s008.doc]

**Table S1: Distribution of histopathologic types in mammary tumors from p200 CUX1 transgenic mice**

| Types of mammary tumors | p200 CUX1  n = 26 |
| --- | --- |
| Adenosquamous carcinoma | 10 (38.5%) |
| Solid Carcinoma | 3 (11.5%) |
| Carcinoma | 2 (7.7%) |
| Sarcoma | 2 (7.7%) |
| Others | 9 (34.6%) |
