# Supplementary material for: RAS Transformation Requires CUX1-Dependent Repair of Oxidative DNA Damage
Source: PLoS Biol. 2014 Mar 11;12(3):e1001807. doi: 10.1371/journal.pbio.1001807 (PMC3949673; doi:10.1371/journal.pbio.1001807)
Supplement: Table S2 — Primer sequences used for PCR amplification and mutation sequencing analysis. (DOC) [file pbio.1001807.s009.doc]

**Table S2 : Primer sequences used for PCR amplification and mutation sequencing analysis.**

|  | **Primers for PCR amplification** | **Primers for sequencing** |
| --- | --- | --- |
| ***Kras***  (G12/G13/Q61) | (F) GCCTGCTGAAAATGACTGAGTATAAACTT  (R) GCAAATCACACTTATTCCCTACCAGG | (F) GCCTGCTGAAAATGACTG  (R) GACCATAGGCACATCTTCAG |
| ***Hras***  (G12/G13/Q61) | (F) ATTGGCAGCCGCTGTAGAAGCT  (R) CACCATTGGCACATCATCTGAATC | (F) ATTGGCAGCCGCTGTAGAAGCTATG  (R) CGAAGGACTTGGTGTTGTTG |
| ***Nras***  (G12/G13/Q61) | (F) TCTGCGGAGTTTGAGGTTTTTG  (R) ACACTTGTTGCCTACCAGCACC | (R) GGGGACATCATCAGAATCTTTC |
| ***Braf***  (L634/V637) | (F) CAGCACCCACACCTCAACAGC  (R) GAAACCAGCCCGATTCAAGG | (R) CAGAACAATCCCAAACGC |
| ***Braf***  (G501/G503/G506) | (F) GCTGTCTTCGGAAATACCAATCC  (R) TTGAGGTGTGGGTGCTGTCAC | (F) CCTCATTACCTGGCTCACTCACTAAC |
| ***Mek1***  (Q56/K57/D67/P124) | (F) TGCCCAAGAAGAAGCCGAC  (R) TGCCCGCTGACCCCAAAATC | (R) GCATAATCTTGTGCTTCTCC |
| ***PI3KC***  (P539, E542, E545) | (F) AAGGAGGAGCACTGTCCGTTG  (R) GGGTAGGATTTCAGGAATAGTTACGC | (F) GAGTTGGAGTTTGATTGGTTCAGC |
| ***PI3KC***  (H1047) | (F) CACCAAGACCAGAGAGTTTGAGAGG  (R) TGTTCCTGACTGTGCCATCCC | (F) GTGTGCCATTTGTGTTGAC |
| ***Pten***  (R130,R177,R233) | (F) TGCCATCTCTCTCCTCCTTTTTC  (R) CCTCTGGTCCTGGTATGAAGAACG | (F) TGACAGCCATCATCAAAGAG |
| ***APE1*** | (F) GGCACATGAAGAAATTGACCT  (R) CAGTGCTAGGTATAGGGTGAT |  |
| ***ATM*** | (F) CAGGCGAAAAGAATCTGGGG  (R) GCACAAAGTAGGGTGGGAAAGC |  |
| ***ATR*** | (F) TGAAAGGGCATTCCAAAGCG  (R) CAATAGATAACGGCAGTCCTGTCAC |  |
| ***BRCA1*** | (F) TTGGAGTGGAACATTGGGGAGG  (R) AAGTGCTGGGATTACAGGCGTGAG |  |
| ***CHK1*** | (F) CAGGTCTTTCCTTATGGGATACCAG  (R) TGGGGTGCCAAGTAACTGACTATTC |  |
| ***CHK2*** | (F) GCTATTGGTTCAGCAAGAGAGGC  (R) TCAGGCGTTTATTCCCCACC |  |
| ***NEIL2*** | (F) GCAATGGGGCAAGGAAAAAGAAAG  (R) GCTACTCTGGAGGCTGAGATGAAG |  |
| ***NTH1*** | (F) AATGGACTCTTGGTGGGCTTCG  (R) ATTCAACAGGCGTGGCTTCC |  |
| ***OGG1*** | (F) GTTCCTCCAACAACAACAT  (R) AGATGCAGTCAGCCACCTTGG |  |
| ***PARP1*** | (F) CAACTTTGCTGGGATCCTGT  (R) TGTTTCCAAGGGCAACTTCT |  |
| ***Polβ*** | (F) AGACTCTCAACGGGGGAATCAC  (R) GCAGATGGACCAATGCCACTAAC |  |
| ***RPA2*** | (F) GCCTGTTTTCATTTCCCACTTTGG  (R) TTCTTTTCCTCTGCCCCTGGAG |  |
| ***TP53*** | (F) CTACCTCCCGCCATAAAAAACTC  (R) CCCACAACAAAACACCAGTGC |  |
| ***XRCC1*** | (F) GCACTTCTTTCTTTACGGGGAGTTC  (R) ACATACTTCAGGCTTGCGGCAC |  |
| ***CATL*** | (F) TTTTGGCTGTCCTCTGCTTGG  (R) ATGTCACCGAAGGCGTTCATC |  |
