# Supplementary material for: RAS Transformation Requires CUX1-Dependent Repair of Oxidative DNA Damage
Source: PLoS Biol. 2014 Mar 11;12(3):e1001807. doi: 10.1371/journal.pbio.1001807 (PMC3949673; doi:10.1371/journal.pbio.1001807)
Supplement: Table S3 — Sequences used to design pTRIPz shRNA against CUX1. (DOC) [file pbio.1001807.s010.doc]

**Table S3 : Sequences used to design pTRIPz shRNA against CUX1.**

| **Name** | **Cat#** | **Oligo ID** | **Mature sense sequence** | **Mature antisense sequence** |
| --- | --- | --- | --- | --- |
| pTRIPz 453 | RHS4696-99355749  (Open Biosystems) | V2LHS_151077 | GACAGCAACTCCAGCTCAA | TTGAGCTGGAGTTGCTGTC |
| pTRIPz 808 | RHS4696-99698992  (Open Biosystems) | V2LHS_151079 | CAAATACGATGAAGAAACT | AGTTTCTTCATCGTATTTG |
| pTRIPz 5326 | - | - | TCTTCTCGTTTGAAACTTTGAA | TTCAAAGTTTCAAACGAGAAGA |
